# MCM4 is a novel prognostic biomarker and promotes cancer cell growth in glioma

**DOI:** 10.3389/fonc.2022.1004324

**Published:** 2022-11-17

**Authors:** Shu Yang, Yixiao Yuan, Wenjun Ren, Haiyu Wang, Zhong Zhao, Heng Zhao, Qizhe Zhao, Xi Chen, Xiulin Jiang, Lei Zhang

**Affiliations:** ^1^ Department of Neurology, The First People’s Hospital of Yunnan Province, The Affiliated Hospital of Kunming University of Science and Technology, Kunming, Yunnan, China; ^2^ Key Laboratory of Molecular Oncology and Epigenetics, The First Affiliated Hospital of Chongqing Medical University, Chongqing, China; ^3^ Department of Cardiovascular Surgery, The First People’s Hospital of Yunnan Province, The Affiliated Hospital of Kunming University of Science and Technology, Kunming, Yunnan, China; ^4^ Department of Neurosurgery, The First People’s Hospital of Yunnan Province, The Affiliated Hospital of Kunming University of Science and Technology, Kunming, Yunnan, China; ^5^ Department of Urology, The Second Affiliated Hospital of Kunming Medical University, Kunming, China; ^6^ First Department of Neurosurgery, The Second Affiliated Hospital of Kunming Medical University, Kunming, China; ^7^ Kunming College of Life Science, University of Chinese Academy of Sciences, Beijing, China

**Keywords:** glioma, MCM family, prognostic model, biomarker, immune infiltration, diagnosis

## Abstract

**Background:**

Gliomas account for 75% of all primary malignant brain tumors in adults and result in high mortality. Accumulated evidence has declared the minichromosome maintenance protein complex (MCM) gene family plays a critical role in modulating the cell cycle and DNA replication stress. However, the biological function and clinic characterization of nine MCM members in low-grade glioma are not yet clarified.

**Methods:**

In this study, we utilized diverse public databases, including The Cancer Genome Atlas (TCGA), Chinese Glioma Genome Atlas (CGGA), Rembrandt, Human Protein Atlas (HPA), Linkedomics, cbioportal, Tumor and Immune System Interaction Database (TISIDB), single-sample GSEA (ssGSEA), Tumor Immune Estimation Resource (TIMER), Genomics of Drug Sensitivity in Cancer (GDSC) and Cancer Therapeutics Response Portal databases to explore the mRNA and protein expression profiles, gene mutation, clinical features, diagnosis, prognosis, signaling pathway, tumor mutational burden (TMB), immune subtype, immune cell infiltration, immune modulator and drug sensitivity of nine MCMs. Afterward, qRT-PCR was utilized to detect the expression of the MCM family in glioblastoma multiforme (GBM) cell lines. The one-, three-, or five-year survival rate was predicted by utilizing a nomogram established by cox proportional hazard regression.

**Results:**

In this study, we found that nine MCMs were consistently up-regulated in glioma tissues and glioma cell lines. Elevated nine MCMs expressions were significantly correlated with a higher tumor stage, isocitrate dehydrogenase (IDH) mutates, 1p/19q codeletion, histological type, and primary therapy outcome. Survival analyses showed that higher expression of MCM2-MCM8 (minichromosome maintenance protein2-8) and MCM10 (minichromosome maintenance protein 10) were linked with poor overall survival (OS) and progression-free survival (PFS) in glioma patients. On the other hand, up-regulated MCM2-MCM8 and MCM10 were significantly associated with shorter disease-specific survival (DSS) in glioma patients. Univariate and multivariate analyses revealed that MCM2 (minichromosome maintenance protein2), MCM4 (minichromosome maintenance protein 4), MCM6 (minichromosome maintenance protein 6), MCM7 (minichromosome maintenance protein 7) expression and tumor grade, 1p/19q codeletion, age, and primary therapy outcome were independent factors correlated with the clinical outcome of glioma patients. More importantly, a prognostic MCMs model constructed using the above five prognostic genes could predict the overall survival of glioma patients with medium-to-high accuracy. Furthermore, functional enrichment analysis indicated that MCMs principal participated in regulating cell cycle and DNA replication. DNA copy number variation (CNV) and DNA methylation significantly affect the expression of MCMs. Finally, we uncover that MCMs expression is highly correlated with immune cell infiltration, immune modulator, TMB, and drug sensitivity.

**Conclusions:**

In summary, this finding confirmed that MCM4 is a potential target of precision therapy for patients with glioma.

## Introduction

Glioma is one of the most common tumors in the central nervous system that mainly includes brain lower-grade glioma (LGG) and glioblastoma multiforme (GBM) (1). Increasing evidence has demonstrated that the molecular characteristics of gliomas include mutated isocitrate dehydrogenase 1 and 2 genes (IDH1/2) and co-deletion of 1p/19q (2). With various advances in diagnosis and therapies for a low-grade glioma, the mortality rate for glioma remains higher. Recently, molecular biomarkers have been indicated to be helpful in the diagnosis and prognosis of various cancers. Therefore, uncovering the molecular mechanisms underlying the initiation and progression of glioma and identifying highly reliable biomarkers is crucial to improving the diagnosis and treatment of glioma patients.

Mounting evidence has demonstrated that MCMs play a central role in the cell cycle and DNA synthesis (3). Members of the MCM family including MCM2-MCM10, these members are evolutionally and functionally conserved throughout eukaryotes (4). It has been shown that abnormal expression of MCMs was correlated to diverse cancer initiation and progression by regulating the cell cycle and DNA replication stress (5). Overexpression of MCM7 was reported to facilitate cell proliferation and invasion of GBM cells (6). Additionally, MCM5 was highly expressed in renal cell carcinoma (RCC) tissues and the ablation of MCM5 inhibited RCC cell line proliferation and repressed tumor growth (7). A recent study proved that MCM2 and MCM5 might take the prognostic markers for colon cancer (8). In our previous study, we developed a new method called CVAA (Cross-Value Association Analysis), which functions without a normalization and distribution assumption. We applied it to large-scale glioma transcriptome data generated by The Cancer Genome Atlas (TCGA) project, and successfully discovered numerous new differentially expressed genes (DEGs) (9). MCM4 is one of these DEGs. However, the expression profiles, genetic alterations, clinicopathological parameters, diagnosis values, prognostic values, and immune functions of MCM4 glioma remain to be further elucidated.

In the current study, we performed a comprehensive analysis of the expression profiles, genetic alterations, clinical-pathological parameters, diagnosis values, prognostic values, and immune functions of the MCMs family in glioma. Furthermore, we explored CNV, DNA methylation, Kyoto Encyclopedia of Genes and Genomes (KEGG) expression of the MCMs family. Finally, we examined the correlation between MCMs expression and immune cell infiltration, immune modulator, TMB, and drug sensitivity. We also applied qRT-PCR to validate the expression of MCMs in glioma cell lines.

## Materials and methods

### Data collection and bio-informatic analysis

TCGA (https://www.cancer.gov/about-nci/organization/ccg/research/structural-genomics/tcga) is a public data platform containing 30 different cancer types and the clinical information of 11,000 patients. The LGG and GBM sequencing data and the corresponding clinical data of the samples were obtained from the TCGA database. After excluding data with missing clinical information, we obtained the 529 LGG and 174 GBM samples for further analysis. Subsequently, we download GTEx normal brain tissue gene expression data from UCSC Xena (https://xena.ucsc.edu/). Based on this GTEx data, we analyzed the MCMs expression level in normal brain tissues using the R language “ggpubr” package. The expression of MCMs in 703 glioma samples from TCGA and 1152 nontumor brain tissues from GTEx was analyzed. Correlation between the expression level of MCMs and clinical data in glioma, Kaplan-Meier survival curve, and ROC curve analysis in glioma were verified with TCGA data.

The CGGA database contains the bioinformatics data of more than 2,000 glioma samples from China, depicting the genomic and molecular genetic characteristics of glioma patients in China, and has guiding significance on the molecular typing and drug target development of glioma. We downloaded mRNA sequencing data and the corresponding clinical data of glioma patients from the CGGA (http://www.cgga.org.cn). After excluding data with missing clinical information, we obtained 807 glioma samples for further analysis, including 472 males and 335 females. According to the median expression level of MCMs, the expression level of MCMs in glioma samples was divided into the low-expression group and the high-expression group. Kaplan-Meier survival curve was used to analyze the influence of MCMs expression on the survival of glioma patients. Correlation between MCMs expression level and clinical data in glioma patients was detected; the accuracy of the prognosis model for glioma was then assessed using a receiver operating characteristic (ROC) curve. Pearson coefficient analysis was used to screen other genes related to MCMs expression in the TCGA databases. For the intersecting genes, the Database for Linkedomics (http://www.linkedomics.org/login.php) was used to perform KEGG analysis on the top 200 genes positively related to MCMs expression (Pearson r > 0.7, P < 0.001). Rembrandt (http://gliovis.bioinfo.cnio.es/) (10) to examine the expression, clinical information and prognosis of MCMs in glioma. The Human Protein Atlas (HPA) (https://www.proteinatlas.org/) database was utilized to examine the protein of MCMs in glioma (11).

### Function analysis for MCMs in glioma

In the present research, we utilized the linkedomics database (http://www.linkedomics.org/login.php) to obtain the co-expression genes of MCMs in glioma. We took the cluster profile package to examine the function of MCMS in glioma (12, 13). GeneMANIA database (http://genemania.org/) used to constructed gene-gene interaction network of MCMs (14).

### Immune Infiltration and tumor mutation burden (TMB) analysis

TIMER (https://cistrome.shinyapps.io/timer/) (15), an interactive web portal, could perform a comprehensive analysis of the infiltration levels of different immune cells. In this study, we use the TIMER database to explore the correlation between MCMs and diverse immune cell infiltration in glioma. The correlation of MCMs and immune cell infiltration in glioma was analyzed in TIMER. The “Gene” module can investigate the relationship between MCMs expression and immune cell infiltration levels (B cells, CD8+ T cells, CD4+ T cells, neutrophils, macrophages, and dendritic cells) using the TCGA database. We also use the GSVA R package to quantify the glioma immune infiltration of 24 tumor-infiltrating immune cells in tumor samples *via* ssGSEA (16). The TISIDB (http://cis.hku.hk/TISIDB/) database utilized analysis of the expression of MCMs in a different immune subtype of glioma (17). In tumor mutation burden (TMB) analysis, Spearman’s correlation analysis was performed to calculate the correlation between gene expression and TMB and MSI score. A p-value of less than 0.05 was considered statistically significant.

### Analysis of the correlation between MCMs expression and drug sensitivity

We utilized the Genomics of Drug Sensitivity in Cancer (GDSC) (https://www.cancerRxgene.org) and the Cancer Therapeutics Response Portal (http://www.broadinstitute.org/ctrp) databases to analyze the correlation between MCMs expression and drug sensitivity (18, 19).

### Gene mutation analysis and LncRNA/miRNA/mRNA network construction

The cbioportal database (http://www.cbioportal.org/) and GSCA (http://bioinfo.life.hust.edu.cn/web/GSCALite/) are used to analyze the gene mutation, DNA methylation of MCMs in glioma (20, 21). To explore the potential upstream regulation of MCMs in glioma, we used the LnCeVar database (http://www.bio-bigdata.net/LnCeVar/index.jsp) to construct a competing endogenous RNA (ceRNA) network (22).

### Constructs, lenti-viral preparation, and establishment of different cell lines

For shRNA knockdown experiments, independent shRNAs targeting a different region of MCM4 RNA were constructed using a pLKO.1 vector (Addgene), and the oligo sequences were provided in follow. Lenti-viruses were generated according to the manufacture protocol as previously documented (23) and indicated cells were infected by viruses twice with 48 h and 72 h viral supernatants containing 4 μg/mL polybrene, and stable cell lines were established by appropriate puromycin selection. The two independent MCM4 targeting sequences are: shRNA#1, 5’-GGGTGGAGATGGACCGCGGCC-3’; shRNA#2, 5’-GCCTTGATGAAGAAGCAGAAC-3’.

### Cell culture conditions

GBM cells lines (including A172, U87, and U251 cells) were purchased from the cell bank of Kunming Institute of Zoology, and cultured in DMEM medium (Corning) including 10% fetal bovine serum (FBS) and 1% penicillin/streptomycin at 37°C an atmosphere containing 95% air and 5% CO2.

### Quantitative real-time PCR

The qRT-PCR assay was performed as documented (24). For Real-time RT-PCR assay, indicated cells were lysed by RNAiso Plus (Takara Bio, Beijing, China, Cat. 108-95-2). Total RNAs were extracted according to the manufacturer’s protocol, and then reverse transcribed using RT reagent Kit (Takara Bio, Beijing, China, Cat. RR047A; TIANGEN Biotech, Beijing, China, Cat. KR211-02). Real-time PCR was performed by FastStart Universal SYBR Green Master Mix (Roche, Cat. 04194194001; TIANGEN Biotech, Beijing, China, Cat. FP411-02) using an Applied Biosystems 7500 machine. The primer sequences are list follows MCM2-F: ATGGCGGAATCATCGGAATCC, MCM2-R: GGTGAGGGCATCAGTACGC; MCM3-F: TCAGAGAGATTACCTGGACTTCC, MCM3-R: TCAGCCGGTATTGGTTGTCAC;MCM4-F: GACGTAGAGGCGAGGATTCC, MCM4-R: AGAGCAGTTTGACGTGCTTCC; MCM5-F: ATGTCGGGATTCGACGATCCT, MCM5-R: CCAGGTTGTAATGCCGCTTG; MCM6-F: GAGGAACTGATTCGTCCTGAGA, MCM6-R: CAAGGCCCGACACAGGTAAG; MCM7-F: CCTACCAGCCGATCCAGTCT, MCM7-R: CCTCCTGAGCGGTTGGTTT; MCM8-F: AATGGAGAGTATAGAGGCAGAGG, MCM8-R: CAGAAGTACGTTTTCCTGTGGT; MCM9-F: AGCGATCAAGTTACACTGGTTG, MCM9-R: GTCTCAAACAGAGTCATGGCA; MCM10-F: TGTCCCTGCGCTACCAAGA, MCM10-R: GATGAGCTTTTGGGATCTGGAG; β-actin-F: CTTCGCGGGCGACGAT, β-actin-R: CCATAGGAATCCTTCTGACC. The expression quantification was obtained with the 2−ΔΔCt method.

### Colony formation and flow cytometry assays

For colony formation assay, indicated cells were seeded in a 6-well plate (China, NEST, Cat. 703001) with 600 cells per well supplemented with 2 mL cell culture medium, and the cell culture medium was changed every 3 days for 2~3 weeks, and then indicated cells were fixed with 4% PFA and stained with 0.5% crystal violet. Annexin V FITC Apoptosis Detection Kit I (556547, BD, China) was used to evaluate the cellular apoptosis according to the manufacturer’s instructions. For cell cycle analysis experiments, indicated cells were digested and washed with PBS twice and then fixed in 75% alcohol overnight at -20°C. The fixed cells were washed three times and then stained with propidium iodide (PI) staining buffer at room temperature for 30 min in the dark, and then the cell cycle was analyzed by the FACSAria SORP machine (BD, USA).

### CCK8 assay

Cell viability and growth were determined using CCK8 assays in 96-well plates. Cells were transfected with the relevant plasmids culturing for 48 h, followed by incubation with 8 μL CCK8 for 4 h. Absorbance was read at 450 nm using a spectrophotometer.

### Statistical Analysis

For the datasets from the TCGA database, statistical analyses were performed using R (v.3.6.3). The Wilcoxon rank sum test and Chi-square test were used to estimate the association between MCMs and clinical pathologic characteristics. The Kaplan-Meier method was used to calculate glioma patient survival rates. Univariate and multivariate cox analyses were performed to assess the correlation between clinical features and OS, DSS, and PFS. For the data regarding the function of MCMs, GraphPad Prism 7.0 was used for statistical analyses. The Student’s t-test evaluated the statistical significance between groups. The significance of the data between the two experimental groups was determined by Student’s t-test, and multiple group comparisons were analyzed by one-way ANOVA. P < 0.05 (*), P < 0.01 (**) and P < 0.001 (***), were significant.

## Results

### MCMs were highly expressed in glioma

**Figure 1:**
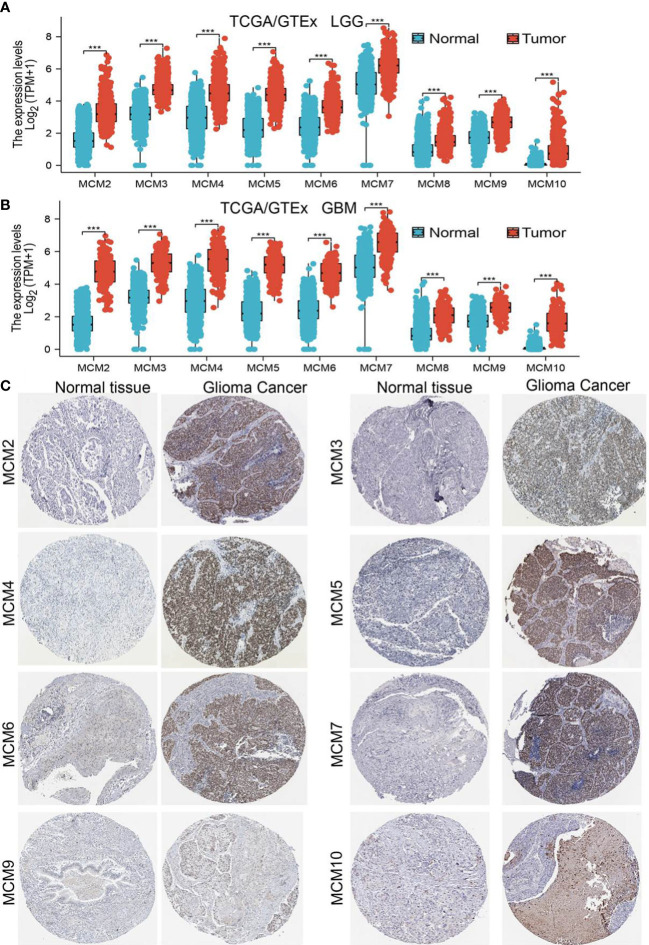
MCMs are highly expressed in glioma. **(A, B)** The expression of MCMs in normal brain tissues and LGG/GBM was examined by TCGA/GTEx databases. **(C)** The protein expression of MCMs in glioma tissue was examined by the HPA database. ***p < 0.001.

**Figure 2:**
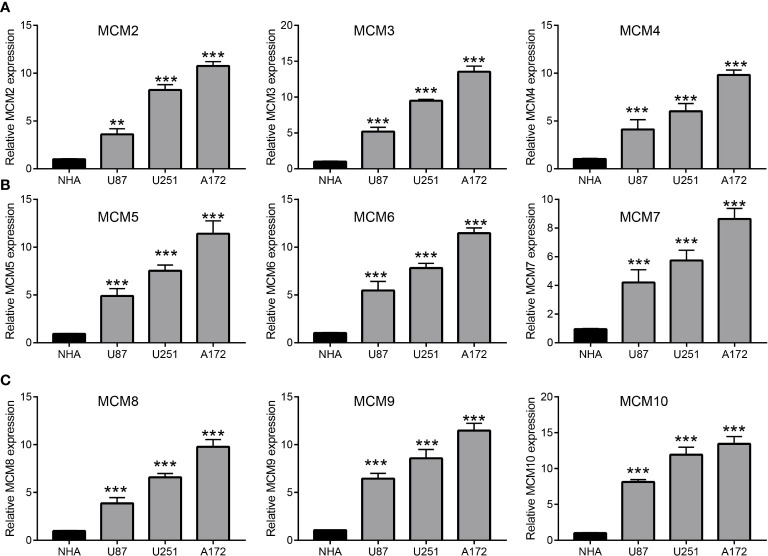
Analysis of the expression of MCMs in glioma cell lines. **(A–C)** The expression level of MCMs in glioma cell lines examine by qRT-PCR assay. **p < 0.01, ***p < 0.001.

We employed TCGA and GTEx databases to examine the mRNA expressions of MCMs in glioma, the results showed nine MCMs were significantly elevated in glioma (**Figures 1A, B**). Furthermore, we uncovered that the protein of nine MCMs was highly expressed in glioma tissue compared with the control group based on the Human Protein Atlas datasets (**Figure 1C**). Finally, we used qRT-PCR assay to verify that nine MCMs were significantly overexpressed in glioma cell lines (**Figures 2A–C**). Collectively, these results suggested that MCMs were high-expressed in glioma tissues and glioma cell lines.

### Relationship between MCMs expression and glioma clinical characteristics

**Figure 3:**
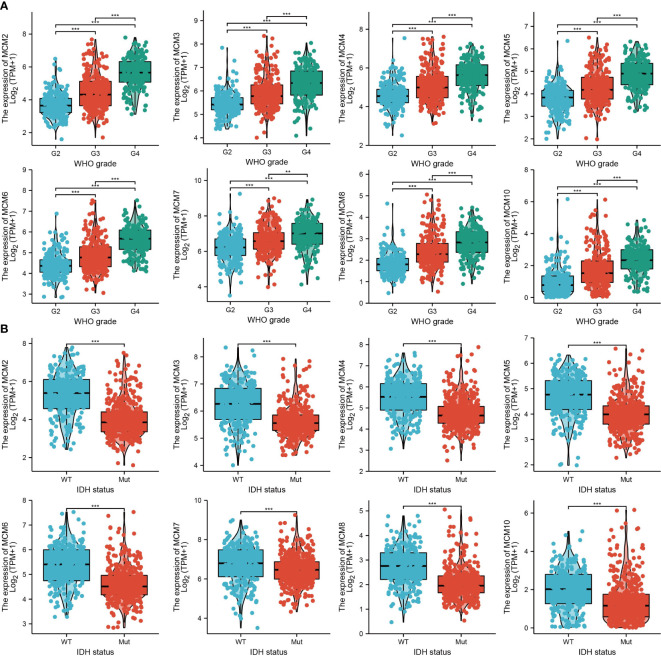
The correlation between MCMs expression and clinical information in glioma. **(A, B)** The correlation between MCMs expression and clinical features, including the higher tumor grades and IDH mutation status in glioma based on TCGA-glioma. **p < 0.01, ***p < 0.001. IDH, Isocitrate dehydrogenase.

**Figure 4:**
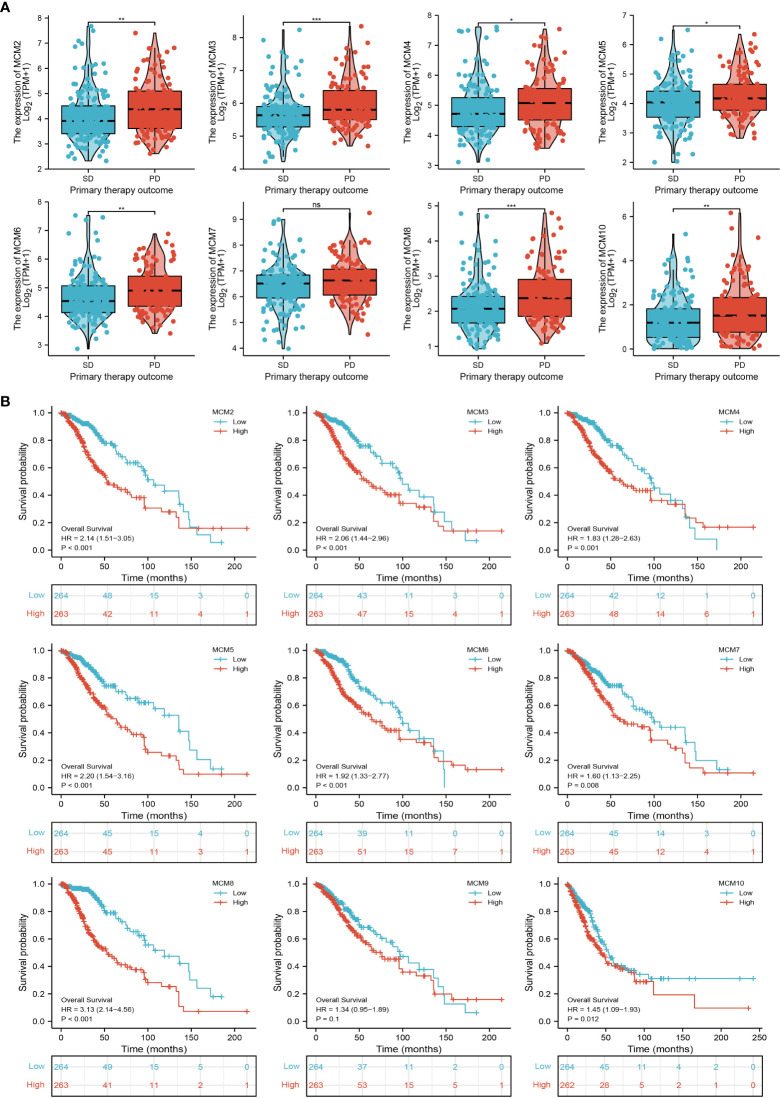
The correlation between MCMs expression and clinical information in glioma. **(A)** The correlation between MCMs expression and clinical features, including the 1p/19q codeletion and histological type in glioma based on TCGA-glioma. **(B)** The overall survival (OS) of MCMS in glioma was examined by the TCGA database. PD, progressive disease; SD, stable disease; NS: p >0.05, *p < 0.05, **p < 0.01, ***p < 0.001.

**Figure 5:**
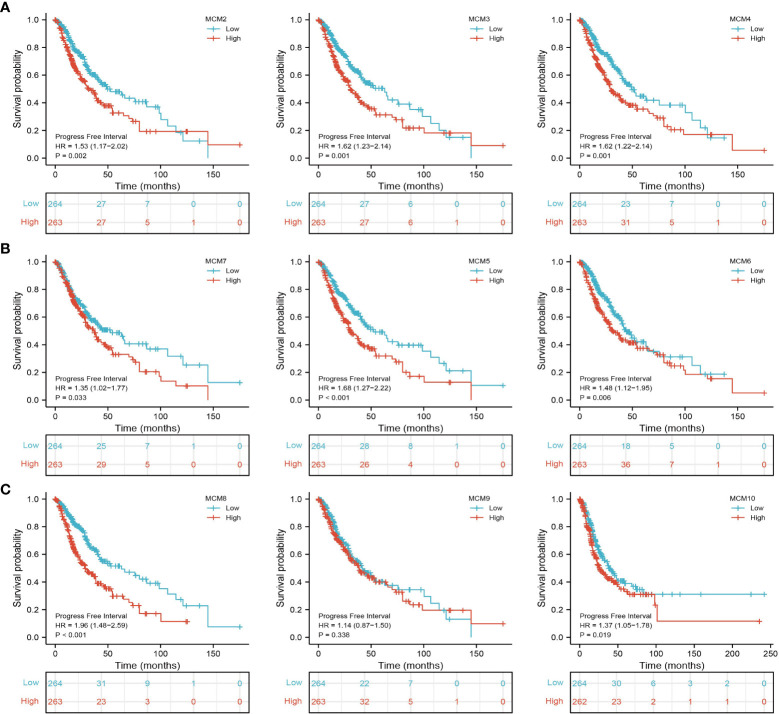
The progression-free survival (PFS) of MCMs in glioma. **(A–C)** The progression-free survival of MCMs in glioma was examined by the TCGA database.

**Figure 6:**
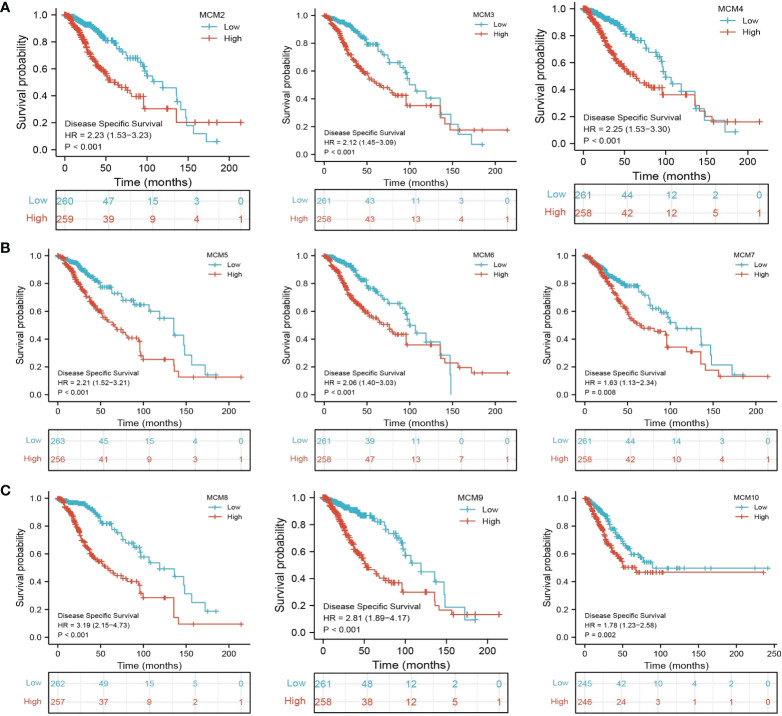
The disease-specific survival (DSS) of MCMs in glioma. **(A–C)** The disease-specific survival and progression-free survival of MCMs in glioma were examined by the TCGA database.

**Figure 7:**
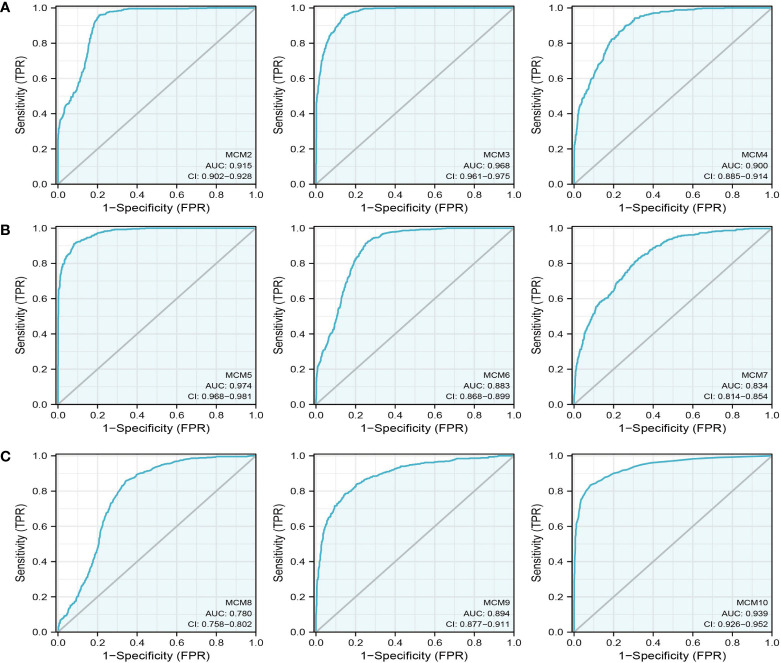
The ROC curve of MCMs in glioma. **(A–C)** The progression-free survival of MCMs in glioma was examined by the TCGA database.

We further explored the correlation between MCMs and clinical characteristics of glioma and uncovered that up-regulated MCM2-MCM8 and MCM10 were significantly associated with the higher tumor stage, IDH mutation status, 1p/19q chromosome co-deletion, histological type, and primary therapy outcome based on the TCGA dataset (**Figures 3**, **4**, **5A**; **Supplementary Figure 1**), this result was verified by CGGA and Rembrandt datasets (**Supplementary Figures 2–5**). Given that MCMs were high-expressed in glioma and their higher expression related to poor clinical characteristics. Therefore, we further explored the prognostic values of MCMs in glioma. glioma patients were divided into high or low-expression groups based on the median expression value. We found that higher expressions of MCM2-MCM8 and MCM10 were linked with poor overall survival (OS) and progression-free survival (PFS) in glioma patients (**Figures 4B**, **5**). On the other hand, up-regulated MCM3-MCM8 and MCM10 were significantly associated with shorter disease-specific survival (DSS) in glioma patients (**Figure 6**). These results were verified by CGGA and Rembrandt datasets (**Supplementary Figures 6, 7**). Given that MCMs differentially expression and are associated with poor clinic-pathologic features and prognosis in glioma. Therefore, we then investigated the diagnosis value of MCMs in glioma. Receiver operator characteristic (ROC) curve analysis results confirmed that MCM2-MCM7, MCM9, and MCM10 had high accuracy (AUC > 0.80) in predicting glioma (**Figures 7A–C**). Univariate and multivariate analyses revealed that MCM2, MCM4, MCM6, MCM7 expression and tumor grade, 1p/19q codeletion, primary therapy outcome, and age were independent factors that influenced the clinical outcome for glioma patients (**Table 1**). The above results confirm that MCMs is a highly sensitive and specific markers with the potential to be used in glioma diagnosis.

**Table 1:** Examine the prognosis of MCMs in glioma patients analysis by cox regression.

Characteristics	Total(N)	Univariate analysis		Multivariate analysis	
		Hazard ratio (95% CI)	P value	Hazard ratio (95% CI)	P value
WHO grade	466				
G2	223				
G3	243	3.059 (2.046-4.573)	<0.001	2.986 (1.578-5.652)	<0.001
1p/19q codeletion	527				
codel	170				
non-codel	357	2.493 (1.590-3.910)	<0.001	3.892 (1.455-10.405)	0.007
Primary therapy outcome	457				
PD	110				
SD	146	0.439 (0.292-0.661)	<0.001	0.361 (0.187-0.697)	0.002
PR	64	0.175 (0.076-0.402)	<0.001	0.261 (0.079-0.868)	0.028
CR	137	0.122 (0.056-0.266)	<0.001	0.210 (0.088-0.501)	<0.001
IDH status	524				
WT	97				
Mut	427	0.186 (0.130-0.265)	<0.001	0.574 (0.258-1.276)	0.173
Histological type	393				
Astrocytoma	195				
Oligodendroglioma	198	0.577 (0.392-0.849)	0.005	0.705 (0.362-1.371)	0.303
Gender	527				
Female	238				
Male	289	1.124 (0.800-1.580)	0.499		
Race	508				
Black or African American	22				
White	486	0.686 (0.319-1.471)	0.333		
Age	527				
<=40	264				
>40	263	2.889 (2.009-4.155)	<0.001	3.734 (2.111-6.606)	<0.001
MCM2	527	1.608 (1.396-1.852)	<0.001	0.546 (0.302-0.989)	0.046
MCM3	527	1.929 (1.585-2.347)	<0.001	0.640 (0.243-1.683)	0.366
MCM4	527	1.722 (1.447-2.051)	<0.001	3.494 (1.580-7.722)	0.002
MCM5	527	1.907 (1.543-2.357)	<0.001	1.717 (0.849-3.473)	0.132
MCM6	527	1.848 (1.517-2.251)	<0.001	2.792 (1.231-6.330)	0.014
MCM7	527	1.350 (1.098-1.660)	0.004	0.503 (0.265-0.953)	0.035
MCM8	527	2.029 (1.702-2.419)	<0.001	0.857 (0.398-1.847)	0.694
MCM9	527	1.704 (1.108-2.621)	0.015	0.471 (0.193-1.148)	0.098
MCM10	527	1.330 (1.180-1.500)	<0.001	0.772 (0.478-1.249)	0.292

WHO, World Health Organization’s; G2, Grade II; G3, Grade III; PD, progressive disease; SD, stable disease; PR, partial response; CR, complete response; WT, Wild Type; Mut: Mutant.

### Prognostic Model Based on MCMs Expression in glioma

**Figure 8:**
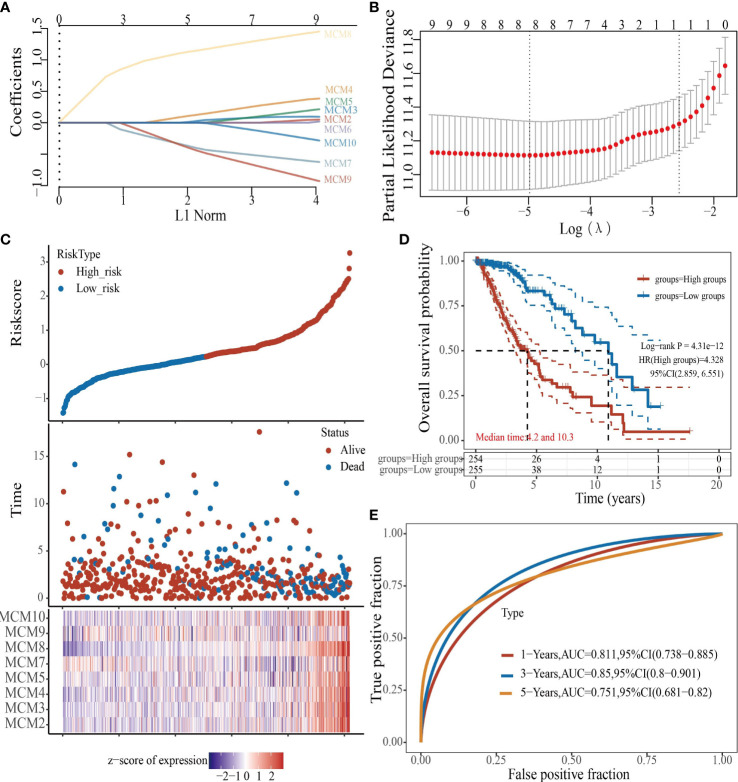
Construction of a prognostic MCMs model in glioma. **(A)** LASSO analysis of the expression pattern of the 9 MCMs. **(B)** Plots of the ten-fold cross-validation error rates. **(C)** Distribution of risk score, state of existence, and MCMs expression in glioma. **(D, E)** OS for glioma patients in the high-/low-risk group and the ROC curve of measuring the predictive value.

**Figure 9:**
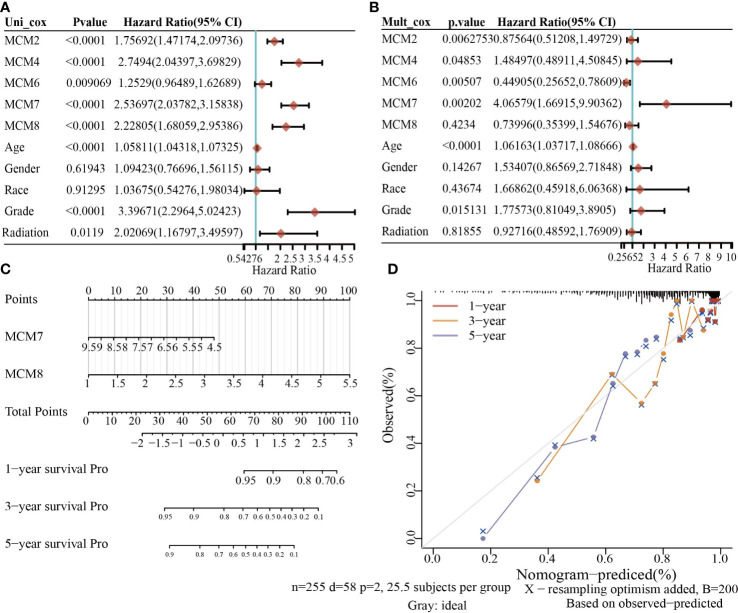
Building the nomogram in glioma. **(A, B)** Hazard ratio and P-value involved in univariate and multivariate cox regression given that clinical feature and five prognostic MCMs in glioma. **(C, D)** Nomogram to predict the 1, 3, and 5-year OS rate of glioma patients.

To construct a prognostic gene model, we constructed a prognostic gene model according to the nine MCMs expressions (**Figures 8A, B**). The risk score= (0.0069)*MCM2+ (0.0927)*MCM3+ (0.3176)*MCM4+ (0.1423)*MCM5+ (0.5626)*MCM7+(1.362)*MCM8+(-0.7806)*MCM9+(-0.1875)*MCM10. According to the risk score, glioma patients were divided into two different groups. With the risk score increasing, the patient’s risk of death and clinical outcome increased and decreased, respectively (**Figure 8C**). Overall survival analysis showed that with high-risk scores glioma patients have a poor clinical outcome (median time = 4.2 years vs. 10.3 years, p <0.0001 (**Figure 8D**), with area under the curve (AUC) values of 0.811, 0.85, and 0.751 in the one, three, and five-year ROC curve, respectively (**Figure 8E**). Given that six prognostic MCMs were correlated with the tumor stage in glioma, our next construction nomogram was used to forecast the overall survival. Univariate and multivariate analyses uncovered that MCM2, MCM4, MCM6, MCM7 expression, tumor grade, and age were independent factors that influence the clinical outcome of glioma patients (**Figures 9A, B**). The predictive nomogram confirmed that the one, three, and five‐year OS rates could be precise predictions (**Figures 9C, D**).

### Validation of the Prognostic Significance of MCMs in diverse glioma cohorts

**Figure 10:**
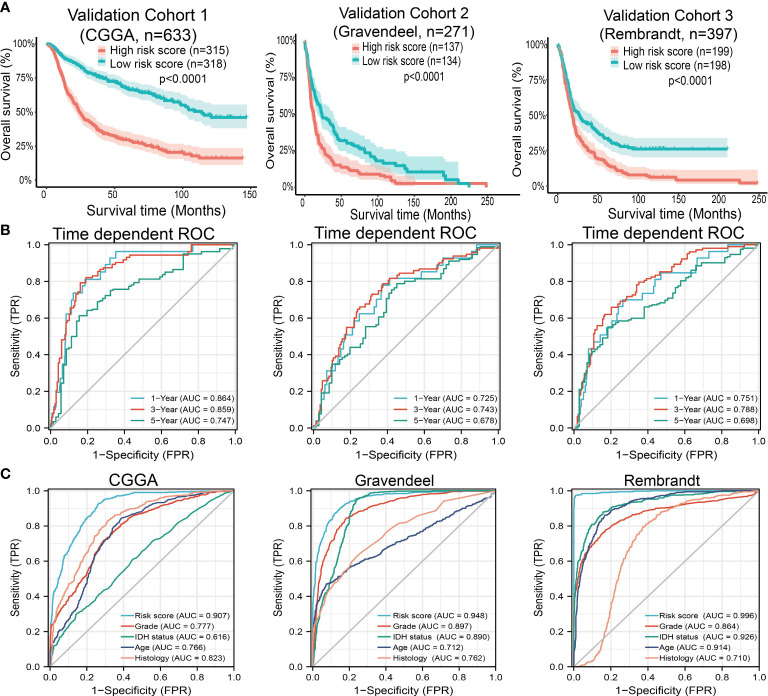
Predictive powers for prognosis of this nomogram. **(A)** Kaplan-Meier survival validation in the CGGA, Gravendeel, and Rembrandt cohorts. Patients with high-risk scores had a poor outcomes in terms of overall survival. **(B)** ROC curves showed the AUC of this nomogram for predicting 1-, 3- and 5-year survival of glioma patients, respectively. **(C)** ROC curves comparing prognostic accuracy of risk score with clinical histology, grade, IDH status, and age in external validation cohorts. AUC, area under the curve; ROC, receiver operating characteristic.

Next, to assess the differences in survival time between low-and high-risk glioma patients, the Kaplan-Meier method was performed. Meanwhile, the log-rank test was also used to determine the statistical significance between groups. Compared with those in the low-risk group, we illustrated that the glioma patients in the high-risk group had shorter overall survival (OS) in the CGGA, Gravendeel, and Rembrandt cohorts, respectively (**Figure 10A**). Finally, the time-dependent ROC curve was also used to assess the predictive power of the nomogram in predicting 1-, 3- and 5-years OS, and the AUC for one-, three- and five-year survival rate of glioma patients was 0.864, 0.859, and 0.8747 in the CGGA cohort, 0.725, 0.743, and 0.678 in the Gravendeel cohort, 0.751, 0.788, and 0.698 in the Rembrandt cohort, respectively (**Figure 10B**). It is noteworthy, compared with the clinical common indicators including WHO grade, IDH mutation, age, and histopathology. This nomogram had a higher predictive power in the CGGA, Gravendeel, and Rembrandt cohorts, respectively (**Figure 10C**). Taken together, these results suggest that this nomogram is a moderately sensitive index for predicting the prognosis of glioma patients, and can act as an effective prognostic biomarker in glioma.

### Gene mutation analysis

We utilized cBioPortal analysis of the genetic alteration of differentially expressed MCMs families. Results confirmed that nine MCMs were all altered, with 0.4, 0.8, 0.4, 1.6, 0.6, 1, 0.6, 0.6 and 1.6 alterations in the glioma samples, respectively (**Supplementary Figure 8A**). We also summarized the incidence of CNV and somatic mutations of nine MCMs in glioma. The results confirmed that missense mutation and C > T were the highest variant type. The findings as well as showed that MCM10 had the highest mutation rate, followed by MCM6 and MCM7 (**Supplementary Figure 8B**). CNV analysis results confirmed that copy number variations of MCM7, MCM9, and MCM3 were significantly positively correlated with its expression in glioma (**Supplementary Figure 8C**). Furthermore, MCM5, MCM8, and MCM10 CNV affected the prognosis of glioma patients (**Supplementary Figure 8D**). Finally, we uncovered that the DNA methylation level of MCM2, MCM5, MCM6, and MCM7 is negatively associated with its expression (**Supplementary Figure 8E**). More importantly, the higher DNA methylation level of MCM2, MCM5, MCM6, and MCM10 were correlated with poor disease-free survival (DSS), OS, and PFS (**Supplementary Figure 8F**).

### Functional analysis of MCMs in glioma

**Figure 11:**
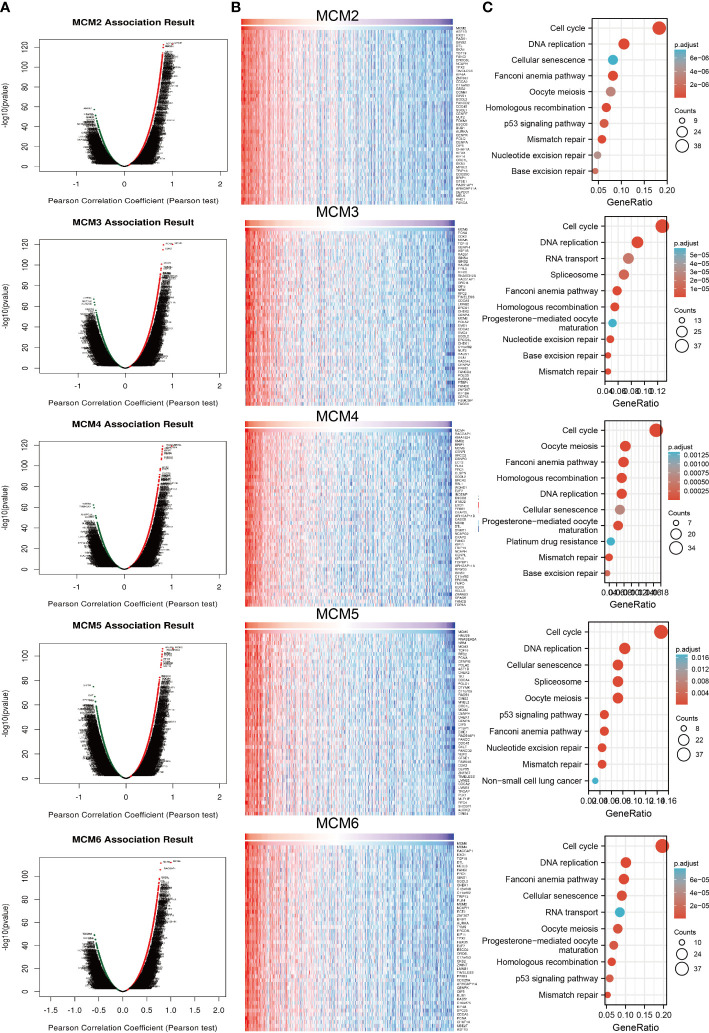
Analysis of the function of MCMs in glioma. **(A, B)** Analysis of co-expression genes of MCMs in glioma by link omics database. **(C)** KEGG enrichment is the signaling pathway of MCMs in glioma. KEGG, Kyoto Encyclopedia of Genes and Genomes.

To explore the functional roles of MCMs in glioma progression, we took linkedomics tools to obtain the co-expression genes that positively correlated with that of MCMs in glioma (**Figures 11A, B**; **Supplementary Figures 9A, B**). Furthermore, we utilized these genes to perform KEGG enrichment analysis. The results confirmed that increased MCMs principal participated in the cell cycle, and cellular senescence (**Figure 11C**; **Supplementary Figure 9C**). Additionally, we uncovered that MCMs showed a high level of activation in the apoptosis, EMT, PI3K/AKT, cell cycle, and DNA damage response (**Supplementary Figures 10A, B**). Finally, we constructed gene interaction networks by the GeneMania database for MCMs. The result confirmed that the most closely related to the MCM genes, including minichromosome maintenance domain containing 2 (MCMDC2), cell division cycle 5-like (CDC5), MCMBP minichromosome maintenance complex binding protein (MCMBP), origin recognition complex, subunit 2 (ORC2), origin recognition complex, subunit 6 (ORC6), GINS complex subunit 2 (Psf2 homolog) (GINS2), TIMELESS interacting protein (TIPIN), claspin (CLSPN), origin recognition complex, subunit 3 (ORC3), origin recognition complex, subunit 4 (ORC4), origin recognition complex, subunit 5(ORC5), GINS complex subunit 1 (Psf1 homolog) (GINS1), cell division cycle 6-like (CDC6), cell division cycle 7-like (CDC7), GINS complex subunit 3 (Psf3 homolog) (GINS3), origin recognition complex, subunit 1(ORC1) and chromatin licensing and DNA replication factor 1 (CDT1) (**Supplementary Figure 10C**). Functional exploration results demonstrated that these genes were correlated with cell cycle regulation. Collectively, these data suggest that MCMs result in glioma cancer initiation and progression by regulating the cell cycle and DNA replication.

### Correlation between MCMs and TMB, immune subtypes of glioma

**Figure 12:**
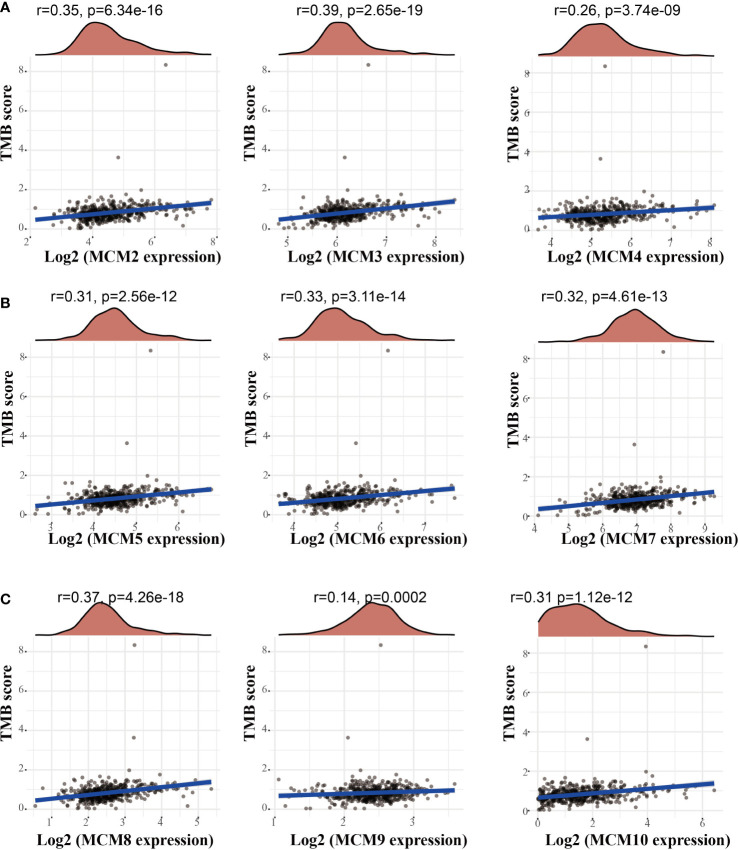
Analysis of the correlation between MCMs and TMB. **(A–C)** Analysis of the correlation between MCMs expression and TMB in glioma. TMB, tumor mutational burden.

Emerging evidence has demonstrated that TMB could be a potential biomarker for predicting the efficacy of immunotherapy for cancer (25). To examine the relationships between MCMs expression and TMB in gliomas. We conducted the related correlation analysis. The findings confirmed that MCMs were positively related to the TMB in glioma (**Figures 12A–C**). Furthermore, we also examined the expression of MCMs in diverse immune subtypes of glioma. The results confirmed that MCMs mainly highly expressed in the C4 subtype (**Supplementary Figures 11A–C**). Collectively, these results indicate that MCMs had different expression patterns in glioma.

### Immune infiltration analysis of the MCMs in glioma

TIMER database analysis was used to explore that somatic copy number alterations of MCMs were significantly related to diverse immune cell infiltration levels in glioma (**Supplementary Figure 12**). Furthermore, we utilized the TIMER database analysis of the correlation between MCMs and diverse immune cells. The results confirmed that MCM2, MCM3, MCM5, MCM8, MCM9, and MCM10 were positively associated with the immune infiltration of B cells, CD4+ T cells, B cells, Neutrophils, Macrophages, and Dendritic. On the contrary, MCM4 is positively related to the CD8+ T cells, Macrophages, and dendritic cells, negatively associated with the immune infiltration of CD4+ T cells. MCM6 is positively correlated with the B cells, CD4+ T cells, and dendritic cells, negatively related to the immune infiltration of CD4+ T cells. MCM7 is positively related to the B cells, and CD4+ T cells, and negatively related to the immune infiltration of CD8+ T cells (**Supplementary Figures 13, 14**). We utilized the cox proportional hazard model and found that macrophages, neutrophils, MCM8, and MCM9 expression were associated with a poor prognosis of glioma.

Finally, we utilized the single sample gene set enrichment analysis (ssGSEA) tools analysis the correlation between MCMs expression and various immune cells. The results confirmed that MCM2 is positively related to the immune infiltration of Th2 cells, T helper cells, and Macrophages, and negatively related to the immune infiltration of NK CD56 bright cells and Tem. MCM3 is positively related to the immune infiltration of Th2 cells, T helper cells, Macrophages, and Th1 cells, and negatively related to the immune infiltration of TFH and NK CD56bright cells. MCM4 positively is related to the immune infiltration of Th2 cells and Treg, and negative related to the immune infiltration of Neutrophils and Macrophages. MCM5 is positively correlated with the immune infiltration of Th2 cells, ADC, and T helper cells, negatively related to the immune infiltration of TFH and NK CD56bright cells. MCM6 is positively related to the immune infiltration of Th2 cells, T helper cells, and Treg, and negatively related to the immune infiltration of NK CD56bright cells and TFH. MCM7 positively is related to the immune infiltration of Th2 cells and pDC, and negative related to the immune infiltration of Th1 cells and Mast cells. MCM8 is positively related to the immune infiltration of Th2 cells, T helper cells, and Ted, and negatively related to the immune infiltration of DC and NK CD56bright cells. MCM9 positively is related to the immune infiltration of Th2 cells, and negatively related to the immune infiltration of NK CD56bright cells. MCM10 is positively related to the immune infiltration of T helper cells and Th2 cells, and negatively related to the immune infiltration of NK CD56bright cells (**Supplementary Figures 15, 16**). Taken together, these results confirm that MCMs play a pivotal role in immune regulation.

### Correlation between MCMs and immune modulators in glioma

Considering immune modulator plays an important role in immune response and development and progression of glioma. We explored the correlation between MCMs and the immune modulator in glioma, including programmed cell death 1 ligand 1 (CD274), cytotoxic T-lymphocyte-associated protein 4 (CTLA4), hepatitis A virus cellular receptor 2 (HAVCR2), lymphocyte-activation gene 3 (LAG3), programmed cell death 1 (PDCD1), programmed cell death 1 ligand 2 (PDCD1LG2), T cell immunoreceptor with Ig and ITIM domains (TIGIT), and sialic acid binding Ig-like lectin 15 (SIGLEC15). The result showed that MMD2, MCM3, MCM5, and MCM8 expression was positively associated with CD274, CTLA4, HAVCR2, LAG3, PDCD1, PDCD1LG12, TIGIT, and SIGLEC15 significantly, while, MCM4, MCM6, and MCM9 were negatively associated with CD274, CTLA4, HAVCR2, PDCD1, PDCD1LG2, TIGIT, and SIGLEC15 significantly (**Supplementary Figure 17**). These results demonstrated that MCMs expression was significantly correlated with the expression of immune modulator-related genes in glioma.

### Correlation between MCMs expression and diverse drug sensitivity

Temozolomide (TMZ) is a first-choice alkylating agent inducted as a gold standard therapy for glioblastoma multiforme (GBM) and astrocytoma. Exploring the potential therapeutic targets is extremely crucial to examine the relationship between these MCMs’ expression and diverse drugs in pan-cancer. In the present study, we utilized the GSCA tools to explore the association between MCMs expression and various drug sensitivity. We found that MCMs were positively correlated with the sensitivity of 17-AAG, RDEA119, selumetinib, and Trametinib, negative correlated with the sensitivity of Genentech Cpd 10, GSK690693, Temozolomide, FK866, CP466722, BMS345541, Vorinostat, NavitoclaxNPK76−II−72−1, Methotrexate, KIN001−102 and AR-42 (**Supplementary Figure 18**).

### Construction of a network of MCMs related to ceRNA

Emerging reports have revealed that the lncRNA/miRNA modulator signaling axis was necessary for gene expression regulation (26). To uncover the upstream regulatory mechanism of MCMs in glioma, we utilized the LnCeVar database identification of MCMs-related ceRNA events in the glioma of patients from TCGA. The results confirmed that the MELTF-AS1/miR-145-5p/miR-1296-5p, PCBP1-AS1/miR-145-5p/miR-1296-5p, H19/miR-145-5p/miR-1296-5p, KDM4A-AS1/miR-145-5p/miR-1296-5p, and RP4-758J18.2/miR-1296-5p regulatory axis may regulate the expression of MCM2, SNHG12/MELTF-AS1/miR-210-3p regulatory axis may regulate the expression of MCM3, EXTL3-AS1/miR-24-3p regulatory axis may regulate the expression of MCM4, SNHG5/miR-885-5p, and AC004893.11/miR-34a-3p regulatory axis may regulate the expression of MCM5, AC012146.7/miR-206, and LINC00869/miR-1-3p regulatory axis may regulate the expression of MCM7, LINC00665/miR-155-5p regulatory axis may regulate the expression of MCM8 and ATP6V0E2-AS1/miR-185-5p, PCBP1-AS1/miR-192-5p/miR-24-3p/, and HCG25/miR-192-5p/regulatory axis may regulate the expression of MCM10 (**Supplementary Figure 19**).

### Knockdown of MCM4 inhibits the cell proliferation of glioma

**Figure 13:**
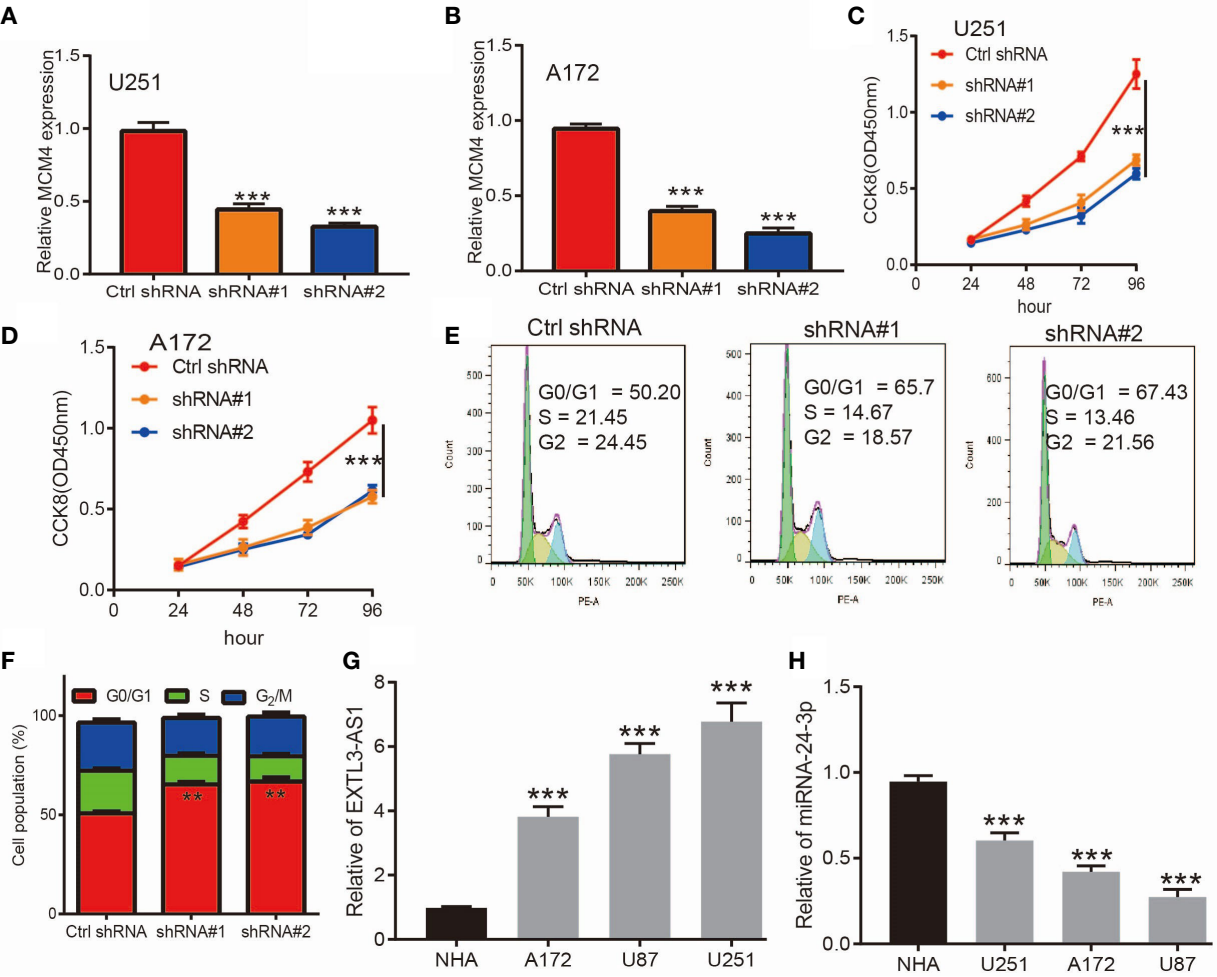
Knockdown of MCM4 inhibits glioma cell proliferation. **(A, B)** The knock-down efficiency of MCM4 in glioma cell lines was examined by qRT-PCR assay. **(C, D)** Depletion of MCM4 inhibits glioma cell proliferation examined by CCK-8 assay. **(E, F)** Depletion of MCM4 increased G0/G1 phase arrested cells. **(G, H)** The expression of EXTL3-AS1 and miRNA-24-3p in glioma cell lines was examined by qRT-PCR assay. shRNA#1=MCM4 shRNA#1, ShRNA#2=MCM4 shRNA#2. **p < 0.01, ***p < 0.001.

Given that MCM4 expression was an independent factor affecting the prognosis of glioma patients and there is no previous study that reported the function of MCM4 in glioma. Therefore, we decided to explore the function of MCM4 in glioma. We firstly we utilized shRNA knockdown of MCM4 in U251 and A172 cells, the knockdown efficiency was verified by qRT-PCR assay (**Figures 13A, B**). The CCK8 assay showed that depletion of MCM4 significantly inhibits glioma cells’ proliferation ability (**Figures 13C, D**). Furthermore, to validate whether MCM4 is critical for cell cycle transition, we performed flow cytometry analysis and revealed that MCM4 knock-down led to increased G0/G1 phase arrested cells (**Figures 13E, F**). We also validated the expression of non-coding RNA that targets MCM4 and found that EXTL3-AS1was highly expressed, and miR-24-3p was down-regulated in glioma cell lines, respectively (**Figure 13G, H**). Collectively, these results demonstrate that MCM4 was highly expressed in glioma cell lines and significantly affected their proliferation and cell cycle.

## Discussion

Glioma is the most common primary tumor of the central nervous system, accounting for 15% of all brain tumors (27). Grades I and II are grouped as low-grade gliomas and grades III and IV as high-grade gliomas, low-grade gliomas have a 10- to 15-year survival. With various advances in diagnosis and therapies for low-grade glioma, the mortality rate for glioma remains higher (28). Therefore, more sensitive and specific diagnostic biomarkers and potential therapeutic targets for this cancer type need to be identified.

Many studies have found that MCM members play an indispensable role in DNA replication (29), embryogenesis (30), maintaining genome instability (31), enhancing cell proliferation (31), and cancer progression (32). For example, overexpression of MCM5 significantly promotes the proliferation and invasion of NSCLC cell lines (33). In particular, MCM2 and MCM6 were reported as potential biomarkers to predict overall survival for LIHC patients (34). Gene mutation of MCM2 was associated with the tumor status, lymph node status, metastatic status, pathologic stage, histologic grade, and prognosis for ESCC patients (35). MCM7 has been reported to be a potential therapeutic target and prognostic biomarker for ESCC patients (36). In this study, we found, for the first time, that MCMs expression was highly upregulated in glioma tissues compared with normal brain tissues. We also showed that MCMs were significantly up-regulated in glioma cell lines, including U251, U87, and A172 cell lines. Meanwhile, elevated MCMs expression was associated with poor clinical characteristics, including higher tumor grades, histological type, IDH mutation status, 1p/19q chromosome co-deletion, and primary therapy outcome. More importantly, we uncover that higher expression of MCM2-MCM8 and MCM10 were linked with poor OS and PFS in glioma patients. On the other hand, up-regulated MCM3-MCM8 and MCM10 were significantly associated with shorter disease-specific survival (DSS) in glioma patients. These results were verified by CGGA and Rembrandt datasets. Receiver operator characteristic (ROC) curve analysis results confirmed that MCM2-MCM7, MCM9, and MCM10 had high accuracy (AUC > 0.80) in predicting glioma. Univariate and multivariate analyses revealed that MCM2, MCM4, MCM6, MCM7 expression and tumor grade, 1p/19q codeletion, primary therapy outcome, and age were independent factors correlated with poor clinical outcomes of glioma patients. These results implied that MCMs overexpression may have played a key role in the malignant phenotypes of gliomas, and is a potentially unfavorable prognostic biomarker for glioma patients.

Given that MCMs expression was correlated with terrible clinical outcomes in glioma. We constructed a prognostic gene model based on 9 MCMs (**Figures 8A, B**). The risk score= (0.0069)*MCM2+ (0.0927)*MCM3+ (0.3176)*MCM4+ (0.1423)*MCM5+(0.5626)*MCM7+(1.362)*MCM8+(-0.7806)*MCM9+(-0.1875)*MCM10. According to the risk score, glioma patients were divided into two different groups. With the risk fraction increasing, the patient’s risk of death and clinical outcome were increased and decreased, respectively. Overall survival analysis showed that with high-risk scores glioma patients have a poor clinical outcome (median time = 4.2 years vs. 10.3 years, p <0.0001, with AUC values of 0.811, 0.85, and 0.751 in the 1, 3, and 5-year ROC curves, respectively. To verify the predictive value of the nomogram, we used diverse glioma cohorts, including the CGGA, Gravendeel, and the Rembrandt cohorts, to assess the differences in survival time between low-and high-risk glioma patients, the Kaplan-Meier method was performed. We found that compared with those in the low-risk group and illustrated that the glioma patients in the high-risk group had shorter OS, respectively. Finally, the time-dependent ROC curve was also used to assess the predictive power of the nomogram in predicting 1-, 3- and 5-years OS, and the AUC for 1-, 3- and 5-year survival rate of glioma patients was 0.864, 0.859, and 0.8747 in the CGGA cohort, 0.725, 0.743, and 0.678 in the Gravendeel cohort, 0.751, 0.788, and 0.698 in the Rembrandt cohort, respectively. It is noteworthy that, compared with the clinical common indicators including WHO grade, IDH mutation, age, and histopathology, this nomogram had a higher predictive power in the CGGA, Gravendeel, and Rembrandt cohorts, respectively. Taken together, these results suggest that this nomogram is a moderately sensitive index for predicting the prognosis of glioma patients, and can act as an effective prognostic biomarker in glioma.

A growing number of studies have reported that CNV and DNA methylation plays an important role in gene expression regulation (37). In this research, we uncovered that copy number variations of MCM7, MCM9 and MCM3 were significantly positively correlated with its expression in glioma. Furthermore, MCM5, MCM8, and MCM10 CNV affect the prognosis of glioma patients. Finally, we uncovered that the DNA methylation level of MCM2, MCM5, MCM6, and MCM7 is negatively associated with its expression. More importantly, the higher DNA methylation level of MCM2, MCM5, MCM6, and MCM10 were correlated with poor DSS, OS, and PFS.

Previous studies reported that MCMs are necessary for cell cycle and DNA synthesis (3). In our study, we found that MCMs principal participated in the cell cycle, DNA replication, p53 signaling pathway, and cellular senescence. Furthermore, we uncovered that MCMs showed a high level of activation in the apoptosis, EMT, PI3K/AKT, cell cycle, and DNA damage response. Emerging evidence has demonstrated that TMB could be a potential biomarker for predicting the efficacy of immunotherapy for cancer (25). In this study, we found that MCMs were positively correlated with TMB in glioma. More importantly, we confirmed that MCMs mainly highly expressed in the C4 subtype of glioma.

It has been shown that CD8+ tumor-infiltrating lymphocytes are related to glioma prognosis (38). Another important finding in the current study is that MCMs expression has a positive correlation with the abundance of infiltrating B cells, CD4+ T cells, CD8+ T cells, macrophages, and neutrophils in glioma. Furthermore, we found that MCM2, MCM3, MCM5, MCM8, MCM9, and MCM10 were positively associated with the immune infiltration of B cells, CD4+ T cells, B cells, Neutrophils, Macrophages, and Dendritic. On the contrary, MCM4 is positively associated with the CD8+ T cells, Macrophages, and Dendritic cells, and negative-positive associated with the immune infiltration of CD4+ T cells. MCM6 is positively associated with the B cells, CD4+ T cells, and dendritic cells, and negatively associated with the immune infiltration of CD4+ T cells. MCM7 is positively associated with the B cells, and CD4+ T cells, and negatively related to the immune infiltration of CD8+ T cells. We utilized the cox proportional hazard model and found that macrophages, neutrophils, MCM8, and MCM9 expression were related to the poor prognosis of glioma patients (**Table 2**). The abundance of immune cells affects the outcomes of patients with head and neck squamous cell carcinoma (HNSCC) and lung adenocarcinoma (LUAD) (39), which is in agreement with the results of this study. As studies have reported, on the one hand, DCs are professional antigen-presenting cells, which can capture and process antigens to present antigenic peptides on MHC Class I and Class II to activate CD8+ and CD4+ cells respectively (40). On the other hand, DCs could promote breast cancer bone metastasis *via* increasing Treg cells and reducing CD8+ cytotoxic T cells and play a crucial role in cell proliferation, invasion, and intercellular communication (41).

**Table 2:** The Cox proportional hazard model of the MCMs family and six tumor-infiltrating immune cells in glioma.

immune cell	coef	HR	95%CI-I	95%CI-U	p-value
B-cell	-1.563	0.21	0	91.952	0.615
CD8_Tcell	-1.284	0.277	0	400.8	0.73
CD4_Tcell	-1.103	0.332	0	1614.2	0.799
Macrophage	8.63	5596.058	89.743	348951	0
Neutrophil	-8.347	0	0	0.531	0.034
Dendritic	1.613	5.019	0.106	237.98	0.413
MCM2	0.195	1.215	0.784	1.883	0.384
MCM3	0.043	1.044	0.512	2.125	0.907
MCM4	0.508	1.662	0.995 ·	2.778	0.052
MCM5	-0.133	0.876	0.482	1.59	0.662
MCM6	0.149	1.16	0.58	2.32	0.674
MCM7	-0.397	0.672	0.427 ·	1.058 ·	0.086
MCM8	1.018	2.767	1.432	5.346	0.002
MCM9	-0.865	0.421	0.218	0.813	0.01
MCM10	-0.218	0.804	0.542	1.192	0.278

In recent years, the study of immune checkpoints has made a breakthrough in the field of tumor immunotherapy, and immune checkpoint blockade has also been approved for the treatment of melanoma and lung cancer (42). Although PD-L1 expression could predict the immunotherapy response of some patients, PD-L1 expression is not enough to predict which patients should be treated with immunotherapy (43). Therefore, an accurate precision biomarker is of great importance to individualized immunotherapy. Interestingly, our results confirmed that MMD2, MCM3, MCM5, and MCM8 expression was significantly positively associated with the CD274, CTLA4, HAVCR2, LAG3, PDCD1, PDCD1LG2, TIGIT, and SIGLEC15, while, MCM4, MCM6, and MCM9 were significantly negatively correlated with CD274, CTLA4, HAVCR2, PDCD1, PDCD1LG12, TIGIT, and SIGLEC15, which suggested that MCMs has the potential to act as a predictive biomarker for the effectors of immune checkpoint blockade in glioma. These findings confirmed that MCMs expression was significantly related to immune modulator-related genes in glioma.

Accumulating evidence suggests vital roles for lncRNA/miRNA in multiple cellular processes and various cancers. Mechanistically, some lncRNA with specific miRNA target sites are capable of regulating gene expression *via* acting as ceRNAs (26). In this study, we constructed the lncRNA -miRNA-mRNA regulatory networks to show the relationships of the MCMs-related lncRNA along with their binding miRNAs and target genes. We uncovered that MCMs were positively or negatively correlated with the diverse drug sensitivity in the cancer therapeutic response portal database.

Minichromosomal maintenance 4 (MCM4) belongs to the family of MCM proteins (MCMs), a group of family proteins strongly linked with DNA replication and cell proliferation, participates in the regulation of DNA replication initiation and can be used as an effective marker for tumor diagnosis (44). In recent years, many studies have proved that MCMs have the potential as prognostic markers in different tumors. However, it has been found that MCM4 expression is related to other tumors. Kikuchi et al. found that MCM4 may play a pivotal role in the proliferation of small cell lung cancer (SCLC) cells, which can be used as a therapeutic target for some patients with SCLC (45). Huang et al. confirmed that the high expression of MCM4 in esophageal cancer was positively correlated with the pathological grade (46). A study reported that some melanoma patients with increased expression of MCM4 have a poor prognosis (47). In this finding, we found that MCM4 was highly expressed in glioma cell lines and significantly affected their proliferation and cell cycle. We also uncovered that MCMs related ceRNA network in glioma progression. These ceRNA plays a critical role in MCMs expression.

To the best of our knowledge, this is the first study to explore the correlation between MCM4 and glioma. However, there are some limitations to our research. First, our study was based on expression data extracted from TCGA but may be more convincing if supported by a prospective clinical study. Furthermore, the biological functions of MCM4 need to be further explored *in vivo* experiments. In the future, we will pay more attention to the function of MCM4 in tumor progression and tumor microenvironment regulation of glioma. Furthermore, we will perform more *in vivo* and *in vitro* experiments to explore the function and the potential molecular mechanisms of MCM4 in tumor progression and tumor microenvironment regulation of glioma.

## Conclusions

In this study, we comprehensively explored the expression pattern, gene alteration, diagnosis, and prognosis of the MCMs family in glioma, by combining the five MCMs, a risk signature was established and validated to be competent to predict the 1-, 3-, and 5-year survival of glioma patients. Furthermore, we also uncovered a correlation between MCMs expression and TMB, immune subtype, immune modulator, immune cell infiltration, and drug sensitivity. Finally, we revealed that MCMs related ceRNA network in glioma progression. In summary, this finding confirmed that MCM4 is a potential target of precision therapy for patients with glioma.

## Data availability statement

The original contributions presented in the study are included in the article/**Supplementary Material**. Further inquiries can be directed to the corresponding authors.

## Author contributions

SY, YY and WR designed this work and performed a related assay. HW, ZZ, HZ, QZ and XC analyzed the data. LZ and XJ supervised and wrote the manuscript. All authors have read and approved the final version of the manuscript.

## Funding

This work was supported by the China International Medical Foundation: Cerebrovascular Disease Youth Innovation Fund (Grant No.Z-2016-20-2101); The Open Project of The First People's Hospital of Yunnan Province Clinical Medicine Center (Grant No. 2022LCZXKF-XZ01); Graduate Student Innovation Fund, Kunming Medical University (Grant No. 2022B24).

## Acknowledgments

The authors would like to thank the CGGA, REMBRANDT, TCGA, GTEx, and GEO databases for providing the data.

## Conflict of interest

The authors declare that the research was conducted in the absence of any commercial or financial relationships that could be construed as a potential conflict of interest.

## Publisher’s note

All claims expressed in this article are solely those of the authors and do not necessarily represent those of their affiliated organizations, or those of the publisher, the editors and the reviewers. Any product that may be evaluated in this article, or claim that may be made by its manufacturer, is not guaranteed or endorsed by the publisher.

## Glossary

**Table:**

AUC	Area under the curve
CCK-8	Cell Counting Kit-8
CNS	Central nervous system
CGGA	Chinese Glioma Genome Atlas
DEGs	Differentially expressed genes
GEO	Gene Expression Omnibus
GSE	Gene Expression Omnibus Series
GO	Gene Ontology
GSEA	Gene Set Enrichment Analysis
GDSC	Genomics of Drug Sensitivity in Cancer
GTEx	Genotype-Tissue Expression
GBM	Glioblastoma
IC50	Half maximal inhibitory concentration
HR	Hazard ratio
HA	Human astrocytes
HPA	Human Protein Atlas
ICB	Immune checkpoint blockade
KEGG	Kyoto Encyclopedia of Genes and Genomes
OS	Overall survival
qRT-PCR	Quantitative real-time reverse transcription PCR
ROC	Receiver operating characteristic
Tregs	Regulatory T cells
REMBRANDT	Repository for Molecular Brain Neoplasia Data
ssGSEA	Single sample gene set enrichment analysis
TCGA	The Cancer Genome Atlas
TIME	Tumor immune microenvironment
TIMER	Tumor Immune Estimation Resource
TME	Tumor microenvironment
WHO	World Health Organization
