# Supplementary material for: MCM4 is a novel prognostic biomarker and promotes cancer cell growth in glioma
Source: Front Oncol. 2022 Nov 17;12:1004324. doi: 10.3389/fonc.2022.1004324 (PMC9713251; doi:10.3389/fonc.2022.1004324)
Supplement: Supplementary file 1 [file DataSheet_1.docx]

Supplementary Material

**Supplementary Figures**


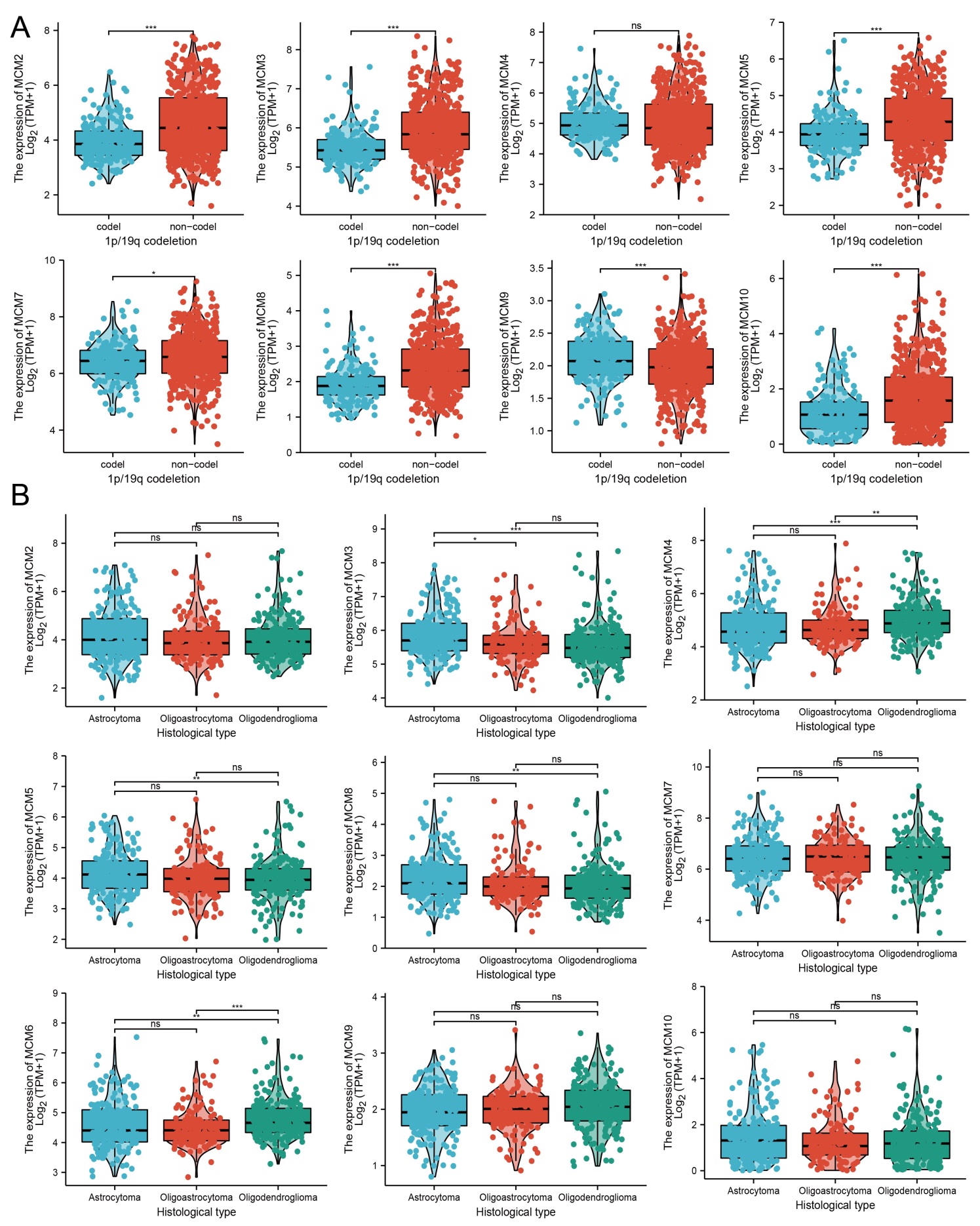


**Supplementary FIGURE 1⎜ The correlation between MCMs and clinical features in LGG.**

(A-B) Analysis correlation between MCMs and clinical features in LGG, including 1p/19q chromosome co-deletion and histological type.

**
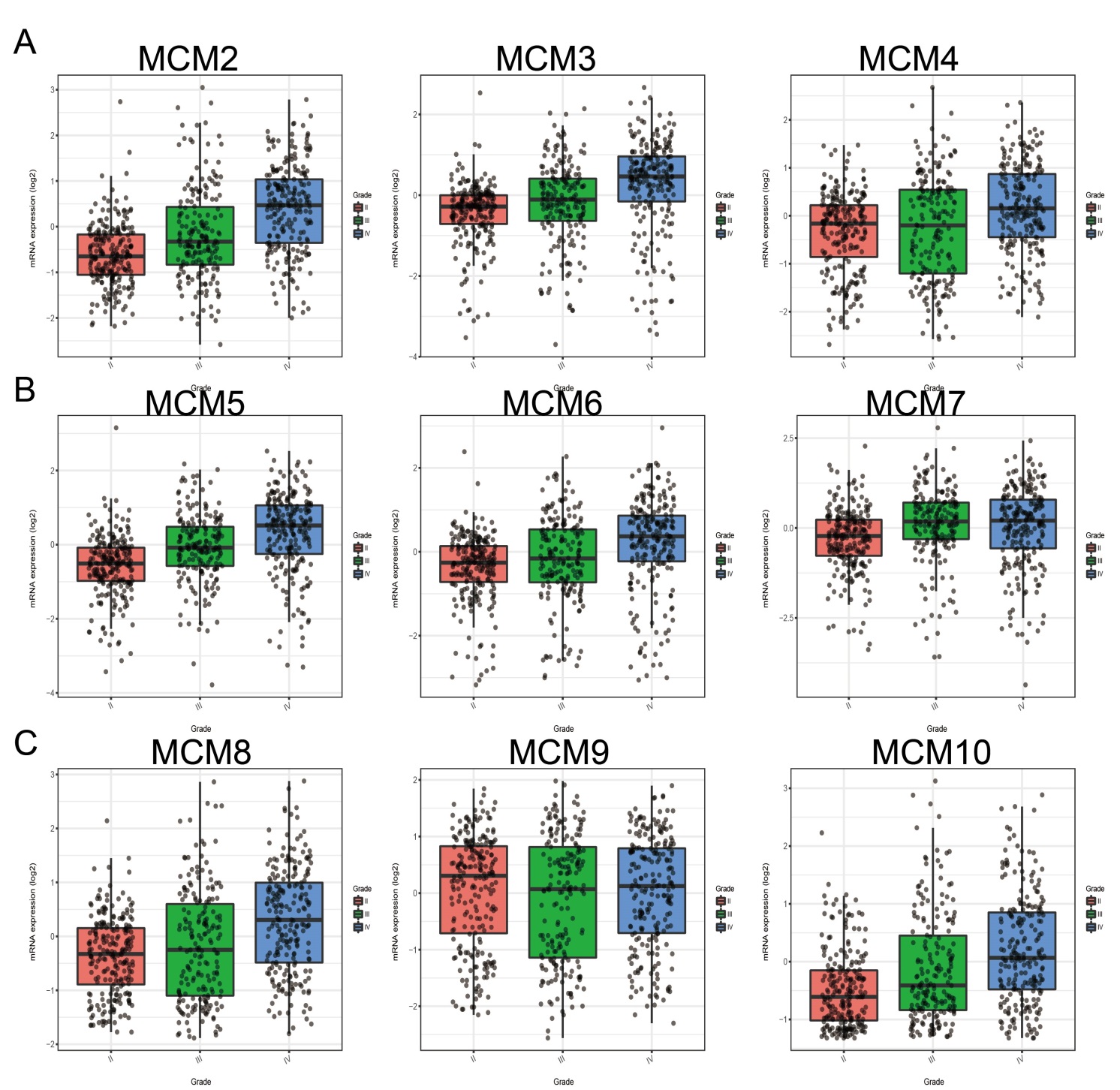
**

**Supplementary FIGURE 2 ⎜ The correlation between MCMs and tumor grade in LGG.**

**(A-C) The correlation between MCMs and tumor grade in LGG by CGGA database.**


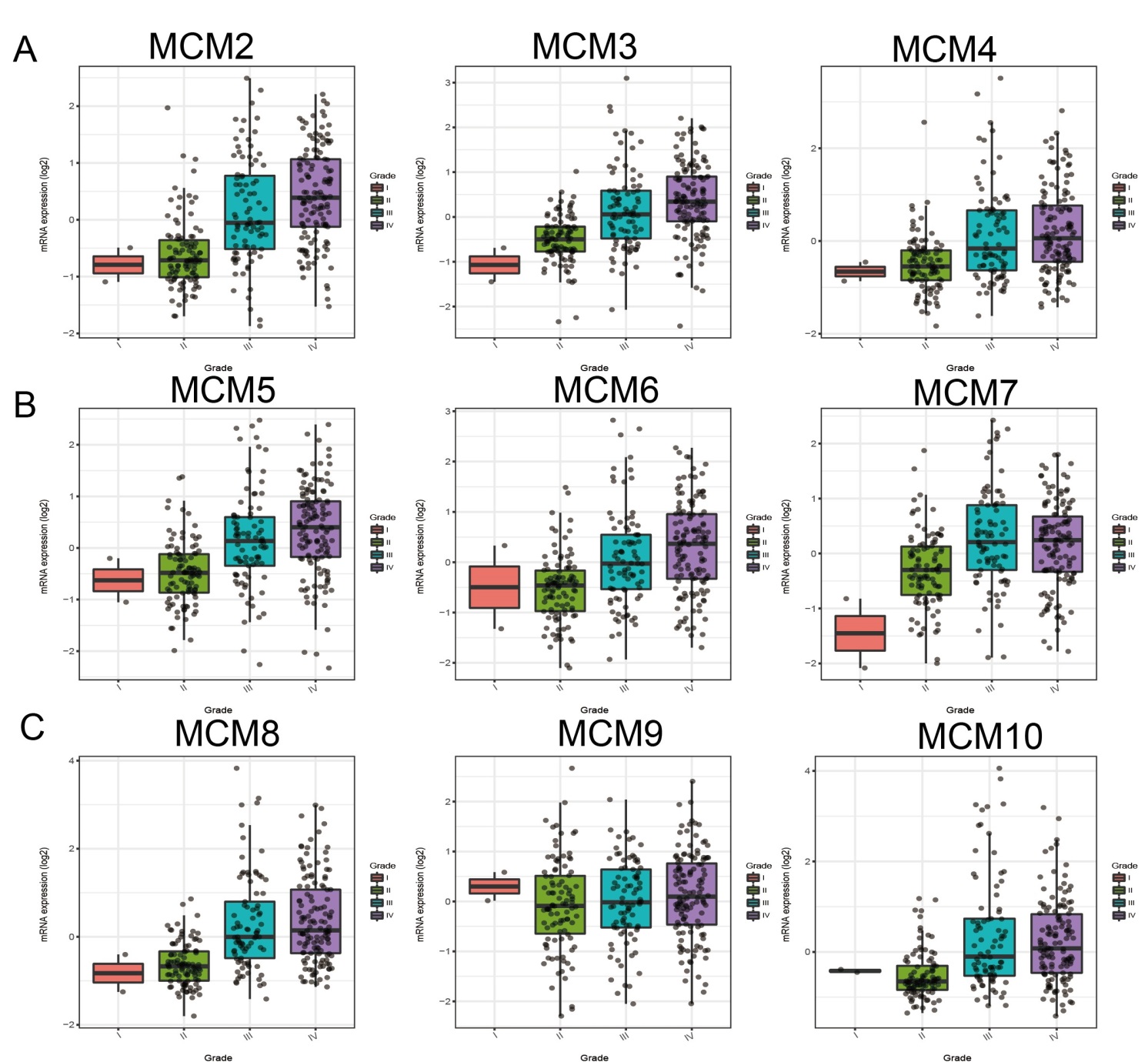


**Supplementary FIGURE 3 ⎜ The correlation between MCMs and tumor grade in LGG.**

**(A-C) The correlation between MCMs and tumor grade in LGG by Rembrandt database.**


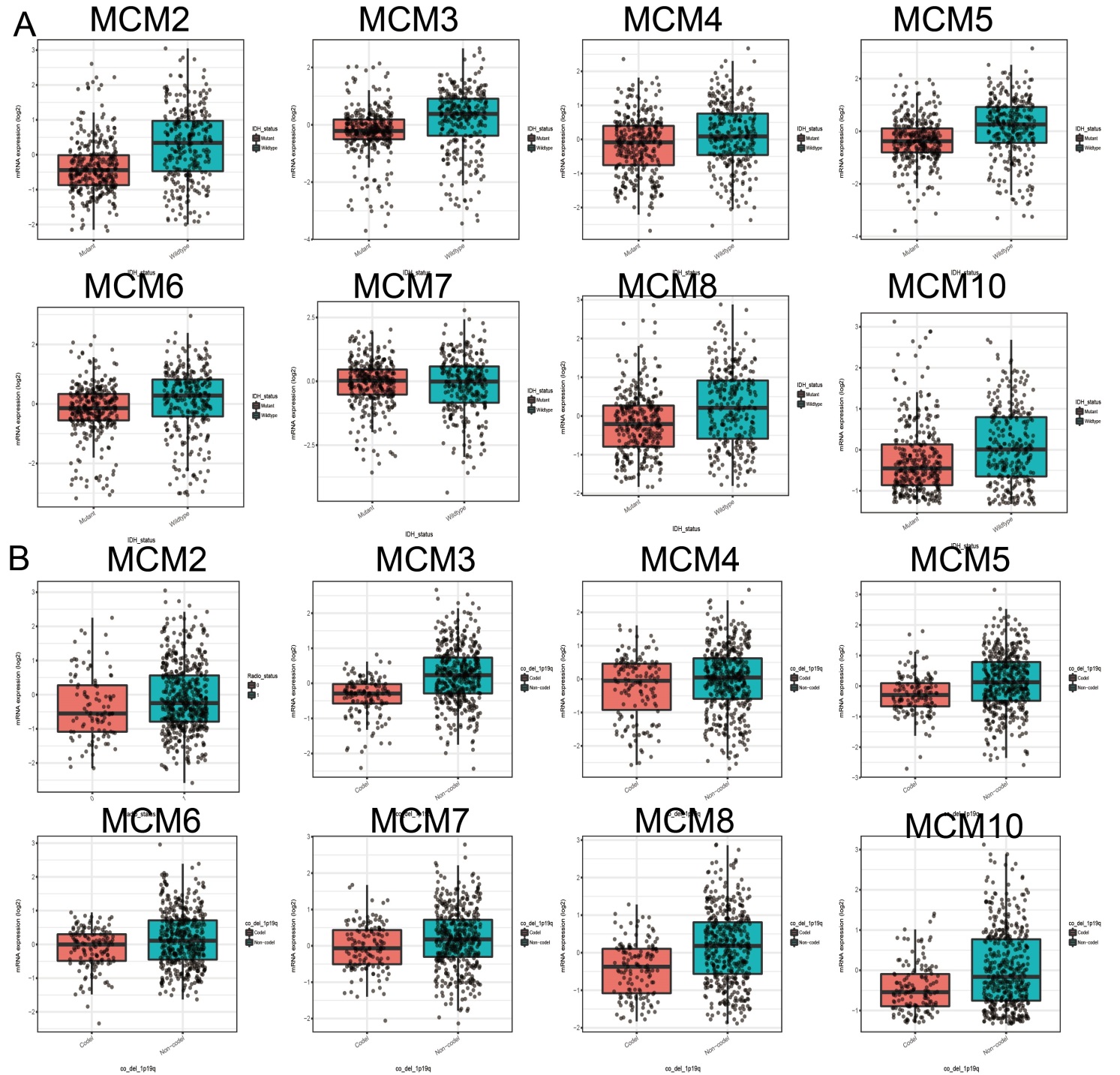


**Supplementary FIGURE 4⎜ Analysis the correlation between the MCMs expression and clinical features in LGG.** (A-B)The correlation between MCMs expression and and clinical features in LGG examine by the CGGA database.. * P < 0.05, ** P < 0.01, *** P < 0.001.


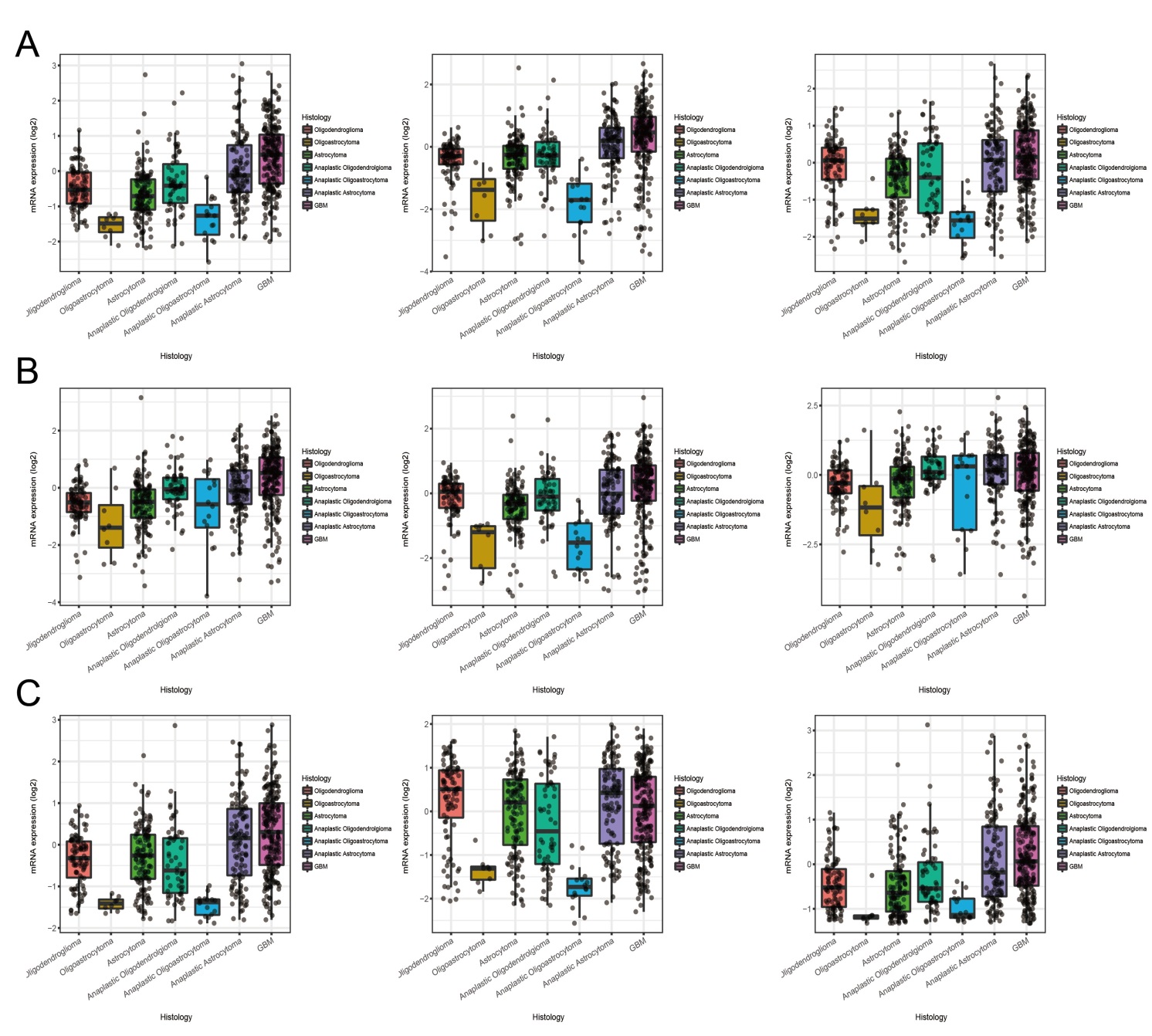


**Supplementary FIGURE 5 ⎜ Analysis the correlation between MCMs expression and histological type in LGG.** (A-C)The correlation between MCMs expression and histological type in LGG examine by the CGGA database. * P < 0.05, ** P < 0.01, *** P < 0.001.


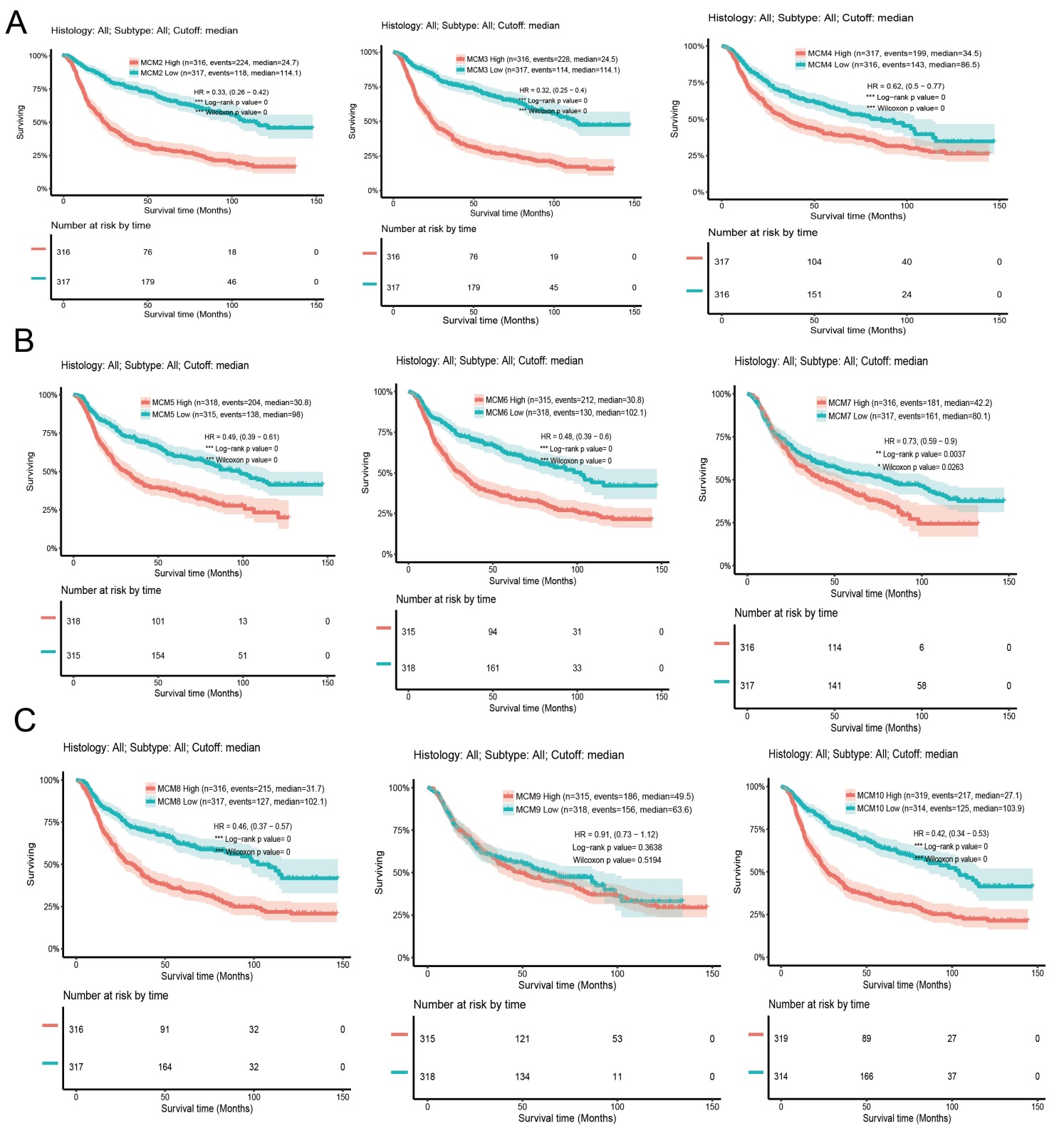


**Supplementary FIGURE 6 ⎜ Analysis the prognosis of MCMs in LGG examine by CGGA database.** (A-C) Analysis the prognosis of MCMs in LGG examine by CGGA database.


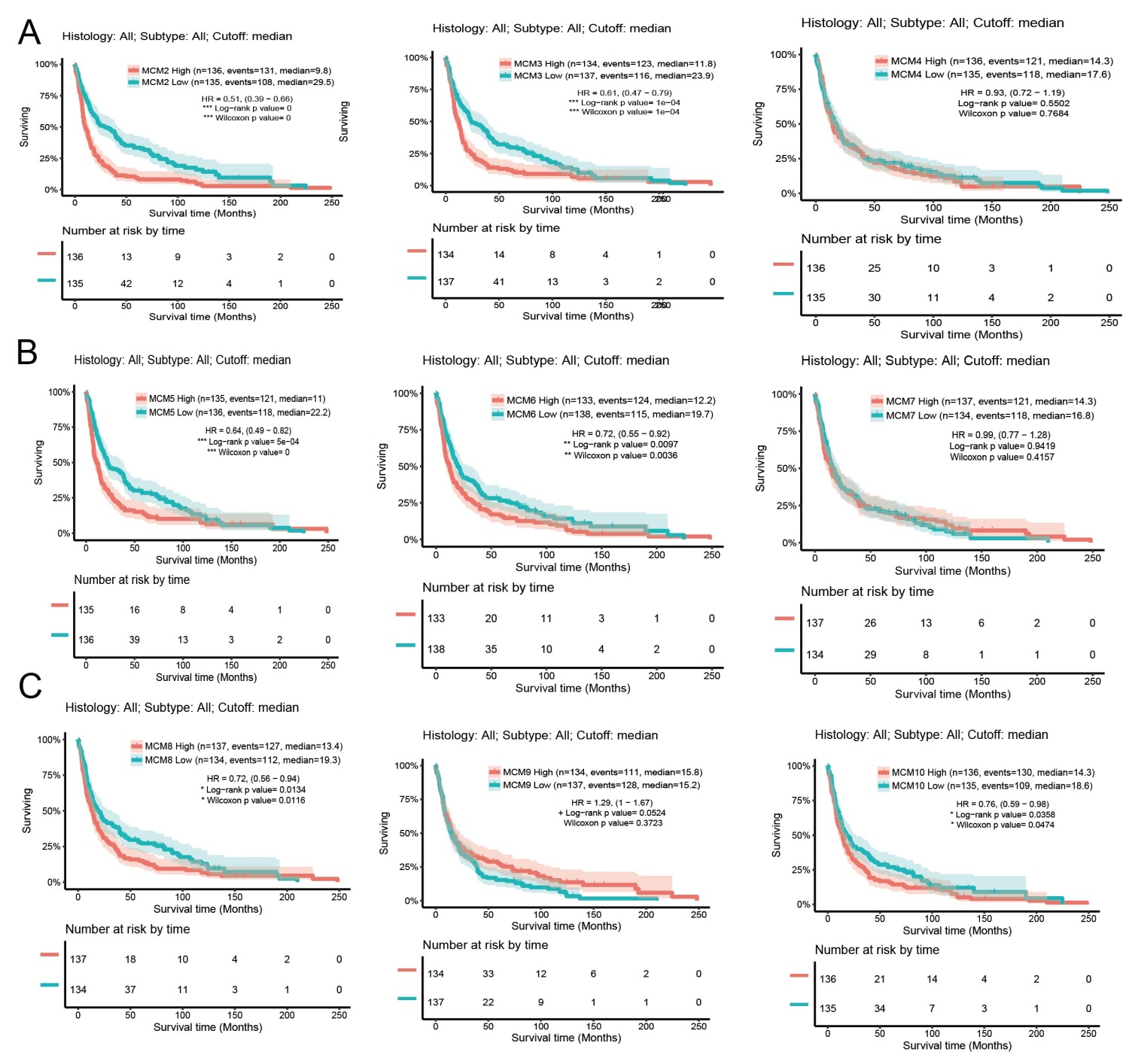


**Supplementary FIGURE 7 ⎜** **Analysis the prognosis of MCMs in LGG examine by Rembrandt database.** (A-C) Analysis the prognosis of MCMs in LGG examine by Rembrandt database.

**
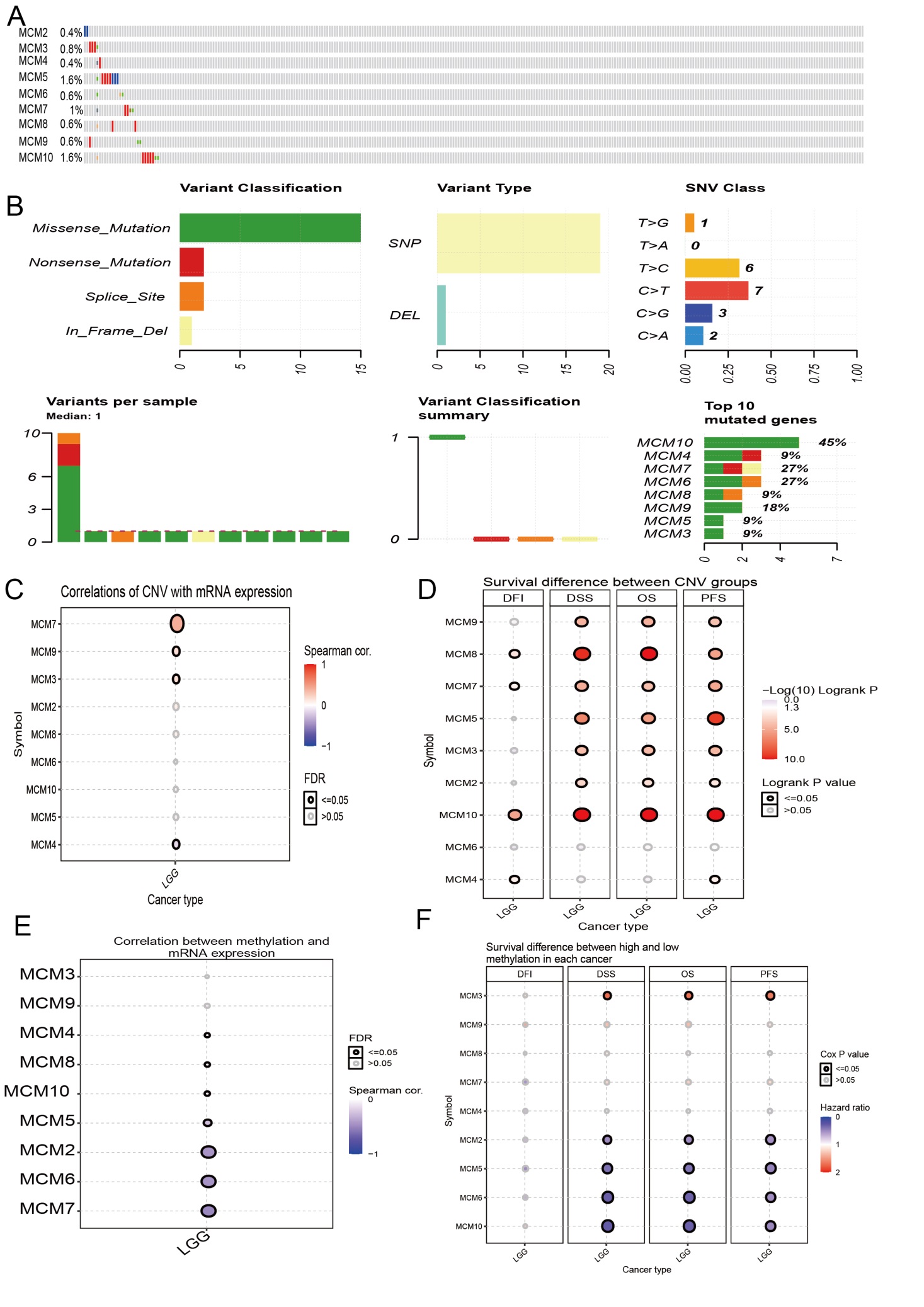
**

**Supplementary FIGURE 8 ⎜Analysis the gene alteration of MCMs in LGG.** (A-B) Analysis the gene alteration of MCMs in LGG by cbioportal database. (C) Analysis the correlation between CNV and its expression. (D) Analysis prognosis of CNV for MCMs in LGG. (E) Analysis the correlation between DNA methylation and its expression. (F) Analysis prognosis of DNA methylation for MCMs in LGG.

**
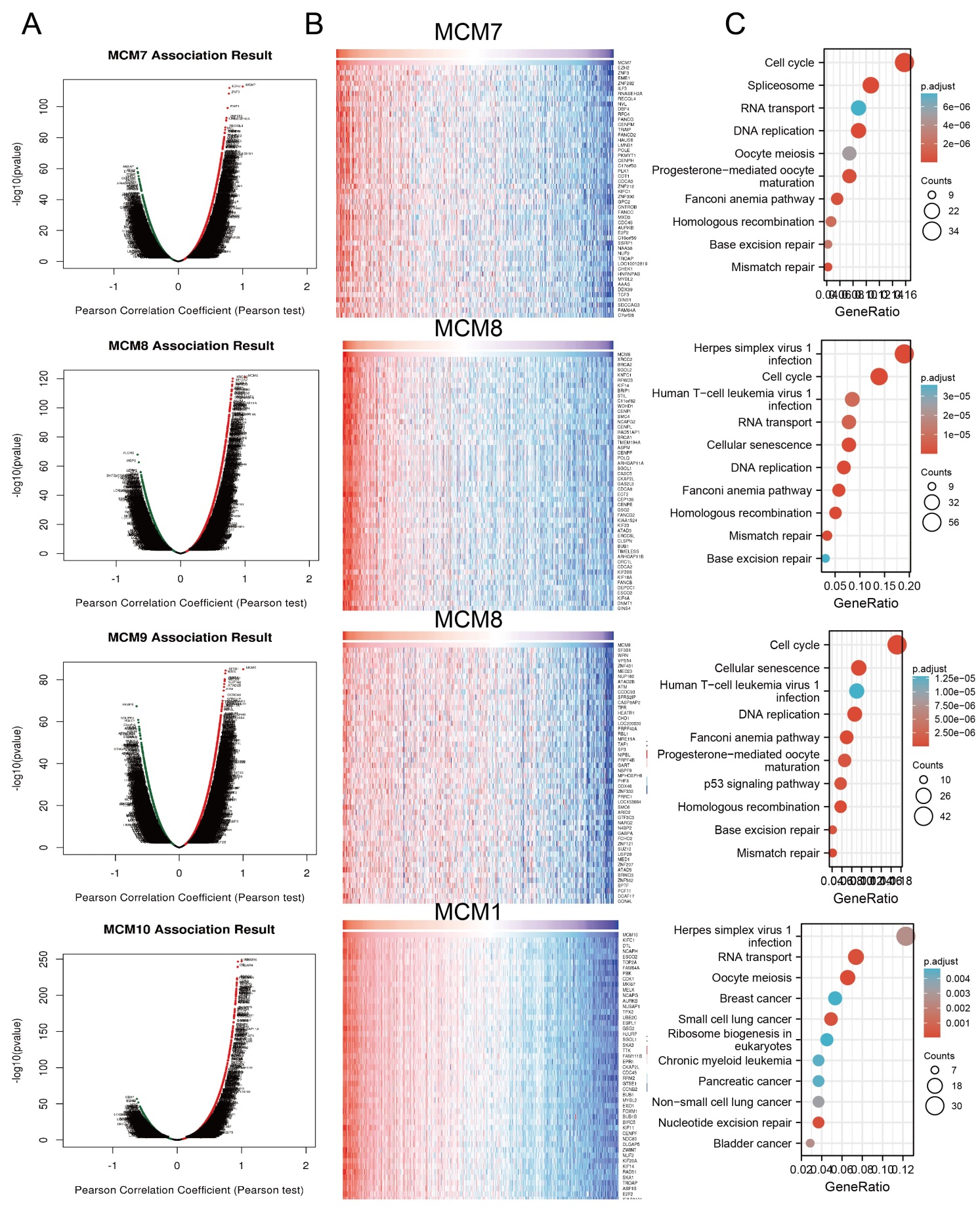
**

**Supplementary FIGURE 9 ⎜ KEGG analysis for MCMs in LGG.** (A-B)The co-expression gene of MCMs in LGG analysed by Linkedomics. (C) KEGG analysis for MCMs in LGG.

**
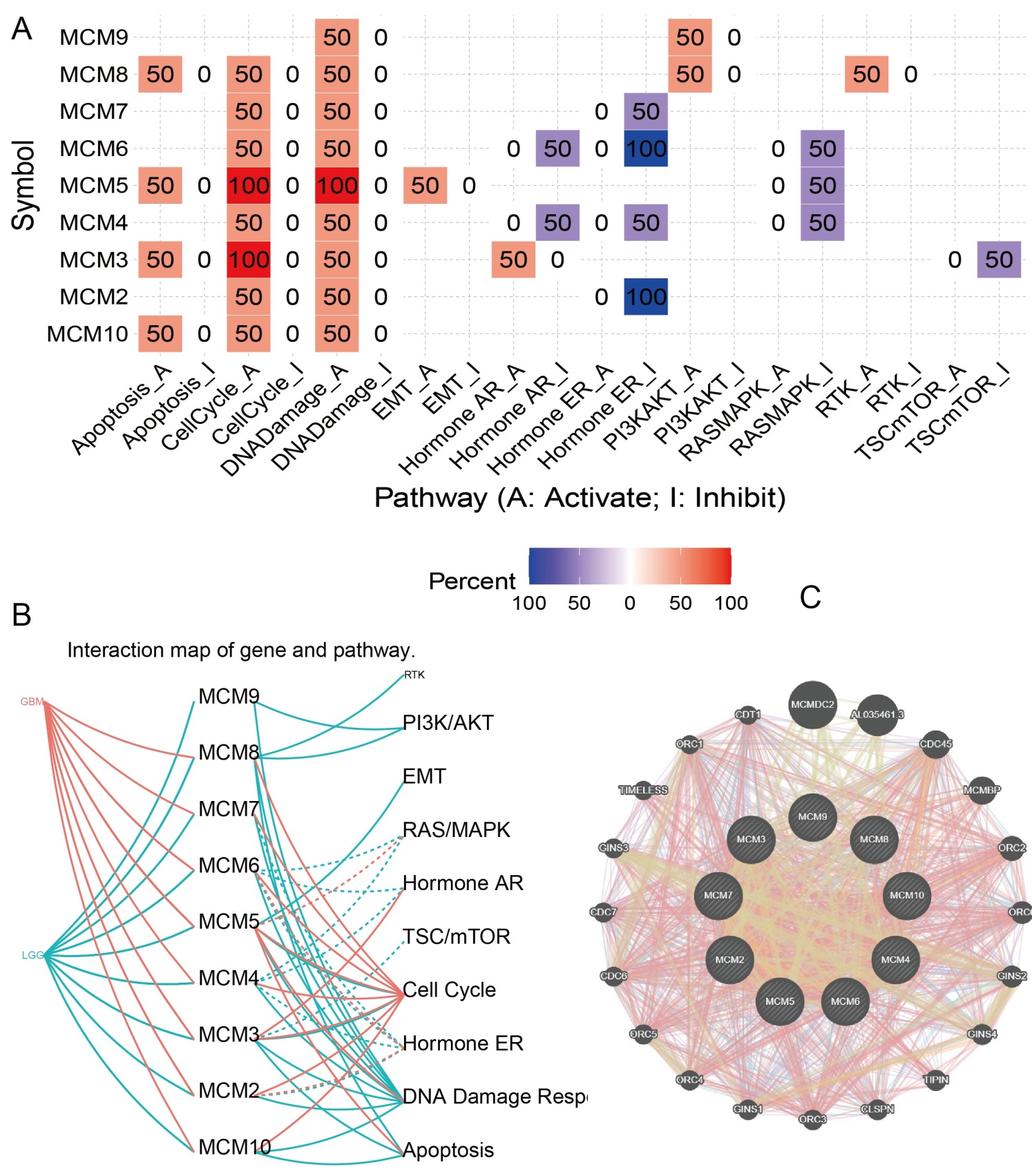
**

**Supplementary FIGURE 10 ⎜Analysis the signaling pathway for MCMs in LGG.** (A-B) The heatmap shows the correlation between the expression MCMs on the important cancer signaling pathways. (C) The gene-gene interaction network of MCMs in LGG analysed by Genemain database.


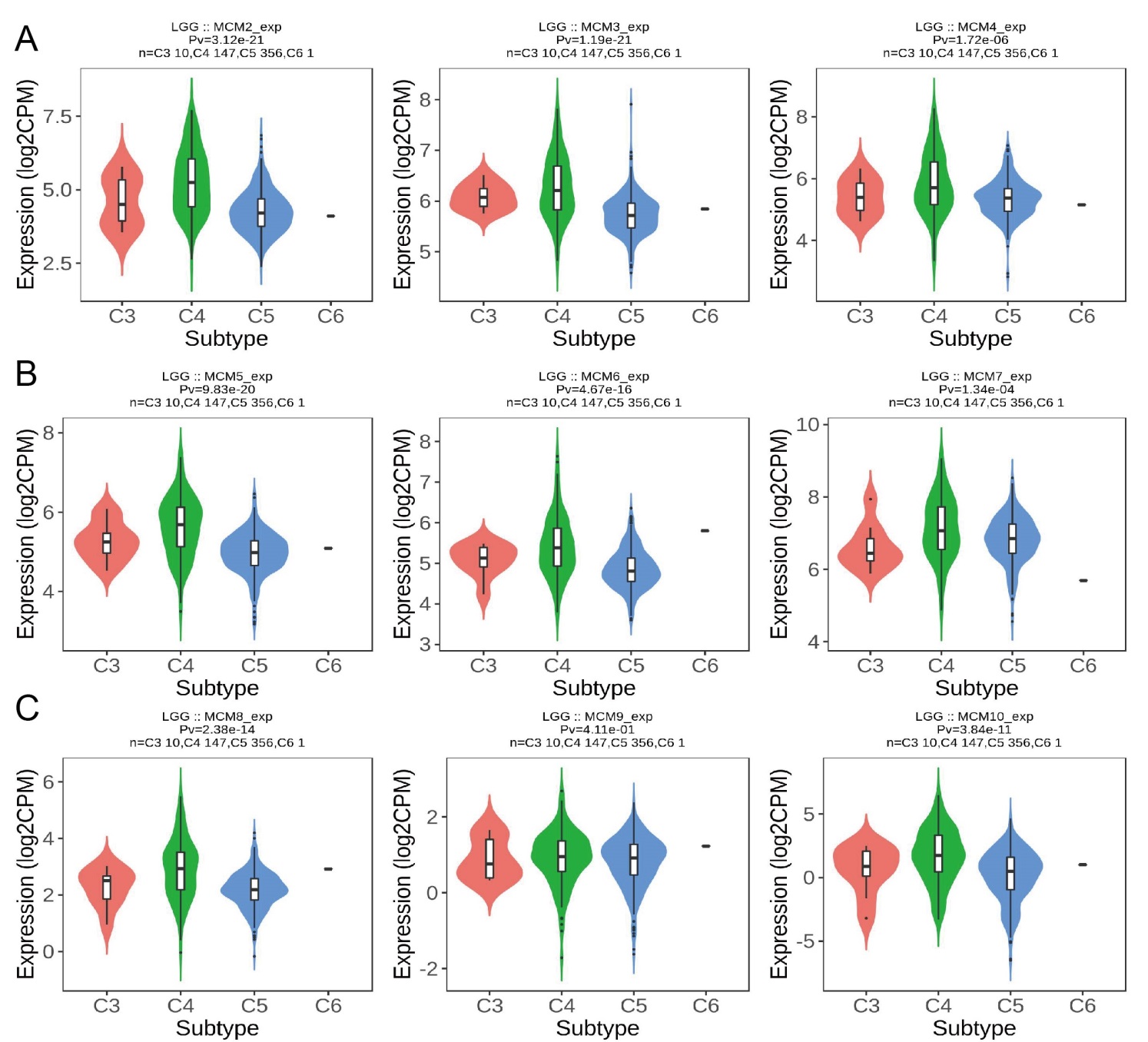


**Supplementary FIGURE 11 Analysis of the expression of MCMs in an immune subtype of glioma.**

**(A-C) Analysis of the expression of MCMs in a different immune subtype of glioma by the TISIDB database.**

**
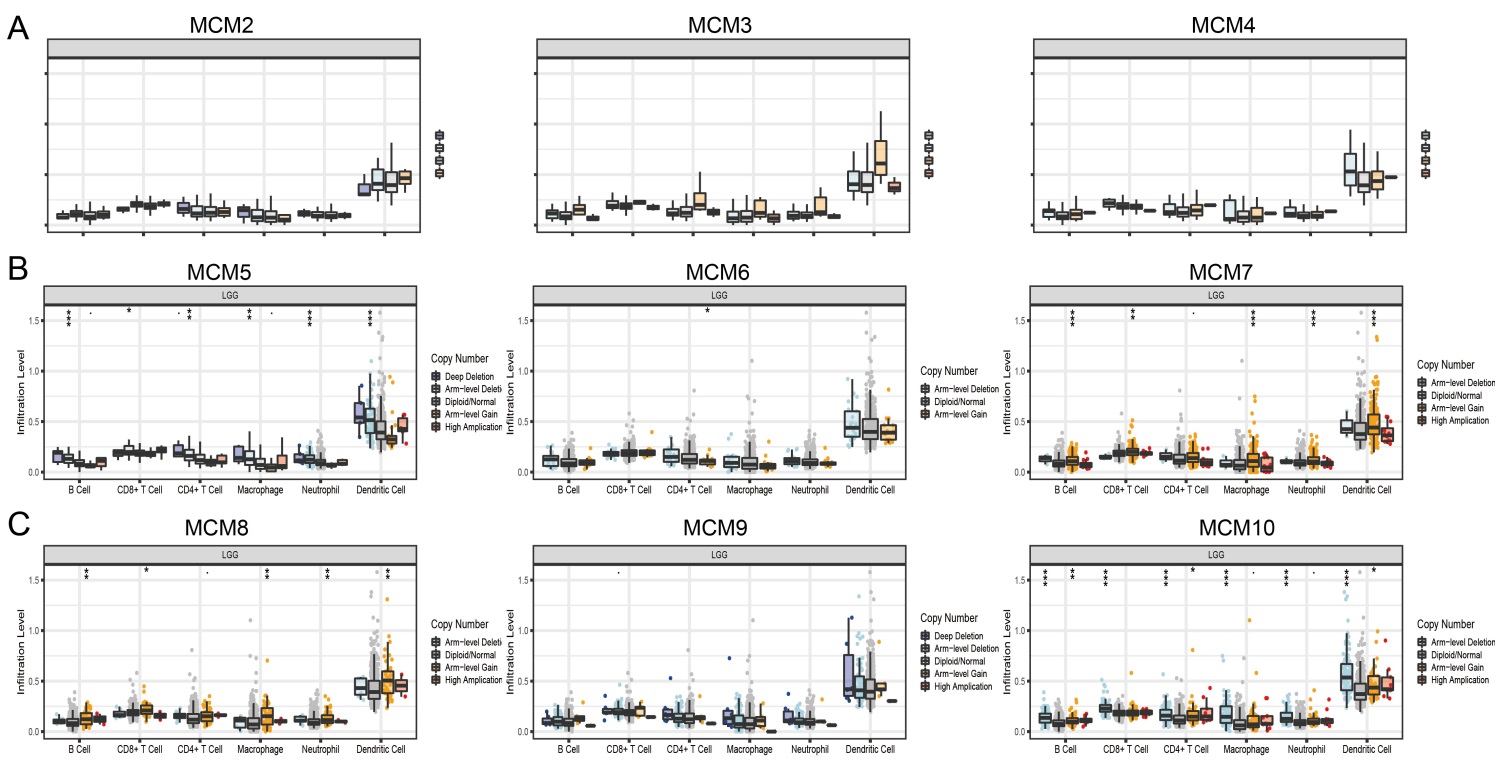
**

**Supplementary FIGURE 12 ⎜** **Analysis the correlation between somatic copy number alterations of MCMs and immune cell infiltration in LGG.** (A-C) Analysis the correlation between somatic copy number alterations of MCMs and immune cell infiltration levels in LGG based on TIMER database.


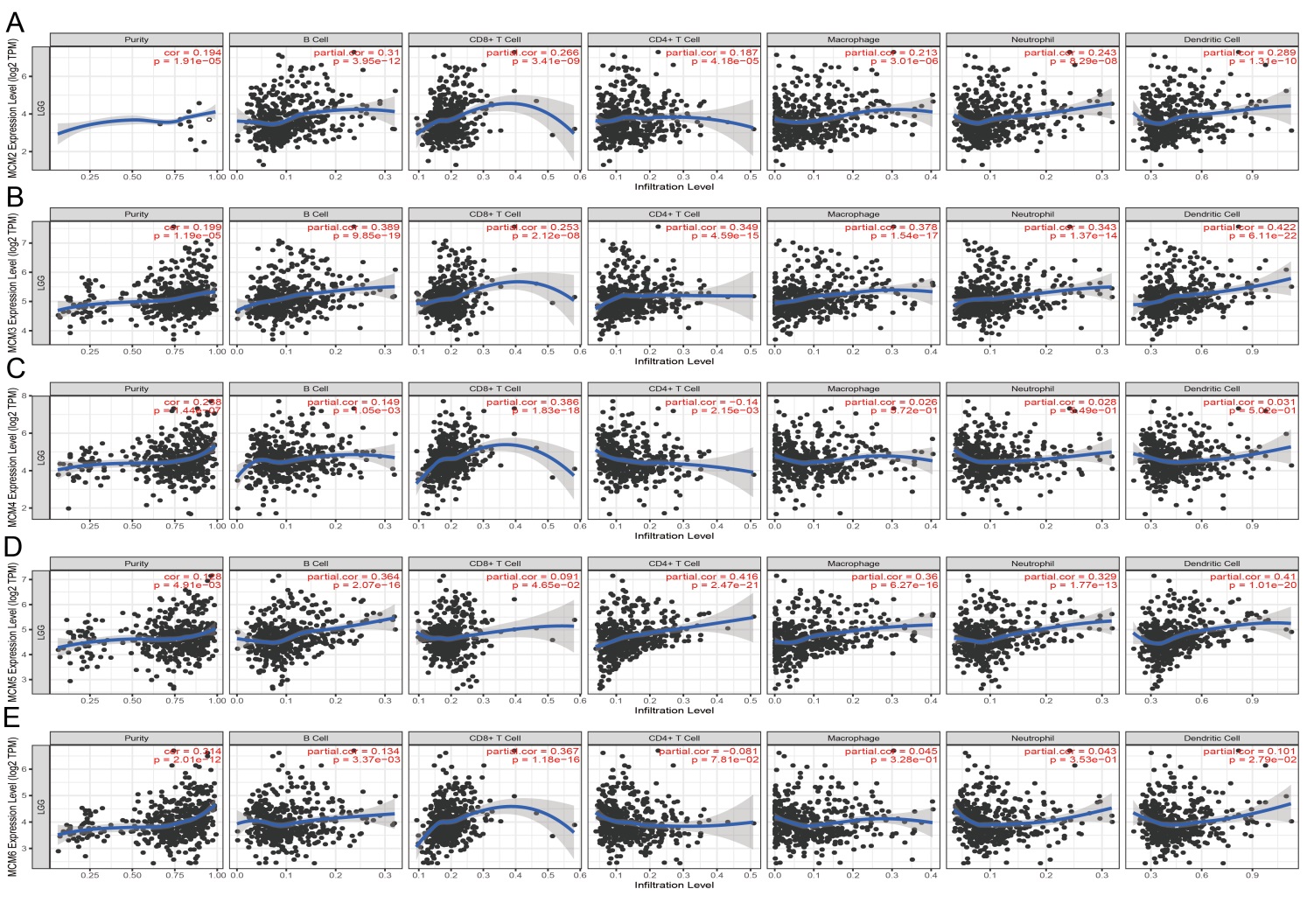


**Supplementary FIGURE 13 ⎜** **Analysis the correlation between the MCMs expression and immune cells infiltration.** (A-E)The correlation between MCMs expression and immune cells infiltration in LGG examine by the TIMER database. * P < 0.05, ** P < 0.01, *** P < 0.001.

**
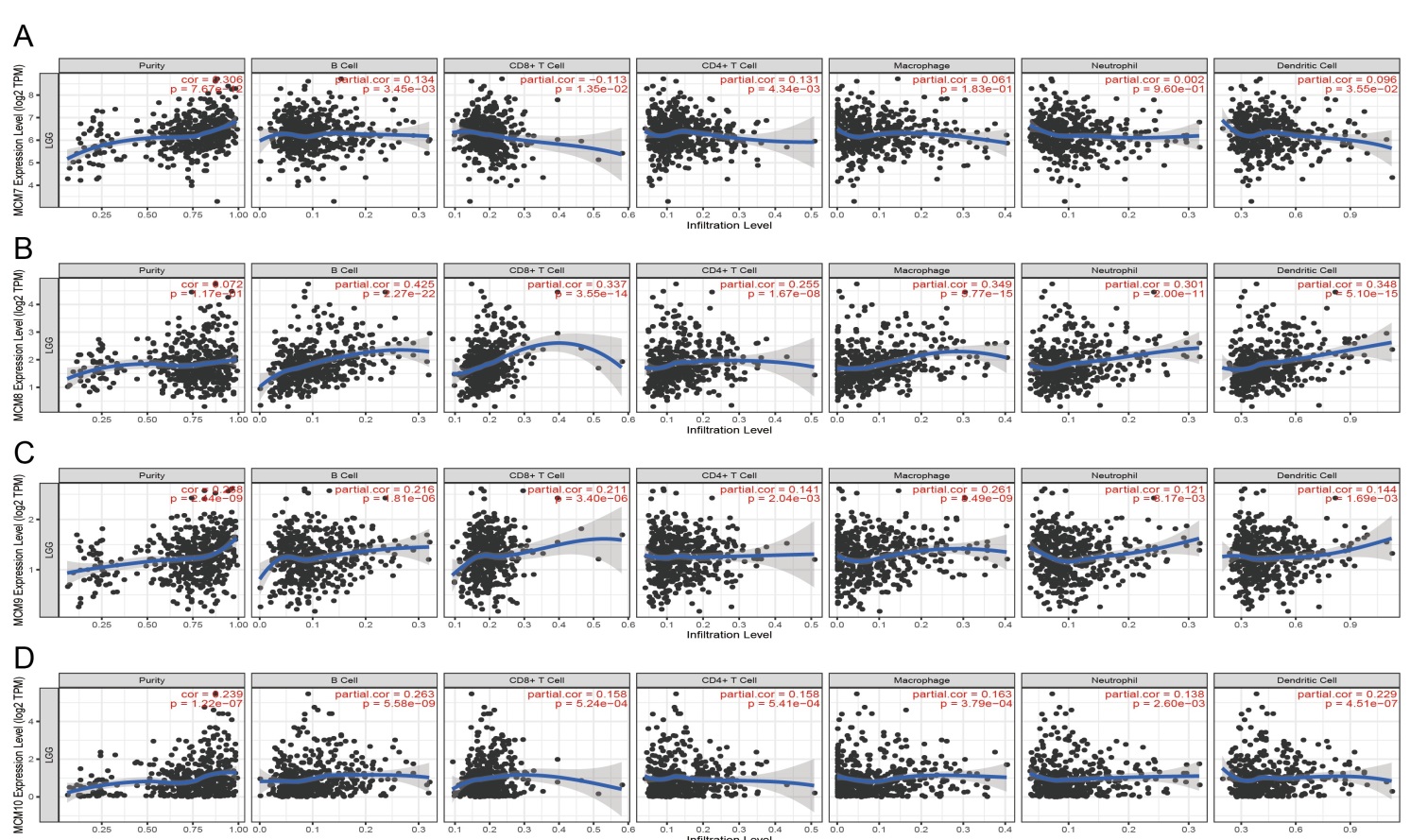
**

**Supplementary FIGURE 14 ⎜** **Analysis the correlation between the MCMs expression and immune cells infiltration.** (A-D)The correlation between MCMs expression and immune cells infiltration in LGG examine by the TIMER database. * P < 0.05, ** P < 0.01, *** P < 0.001.


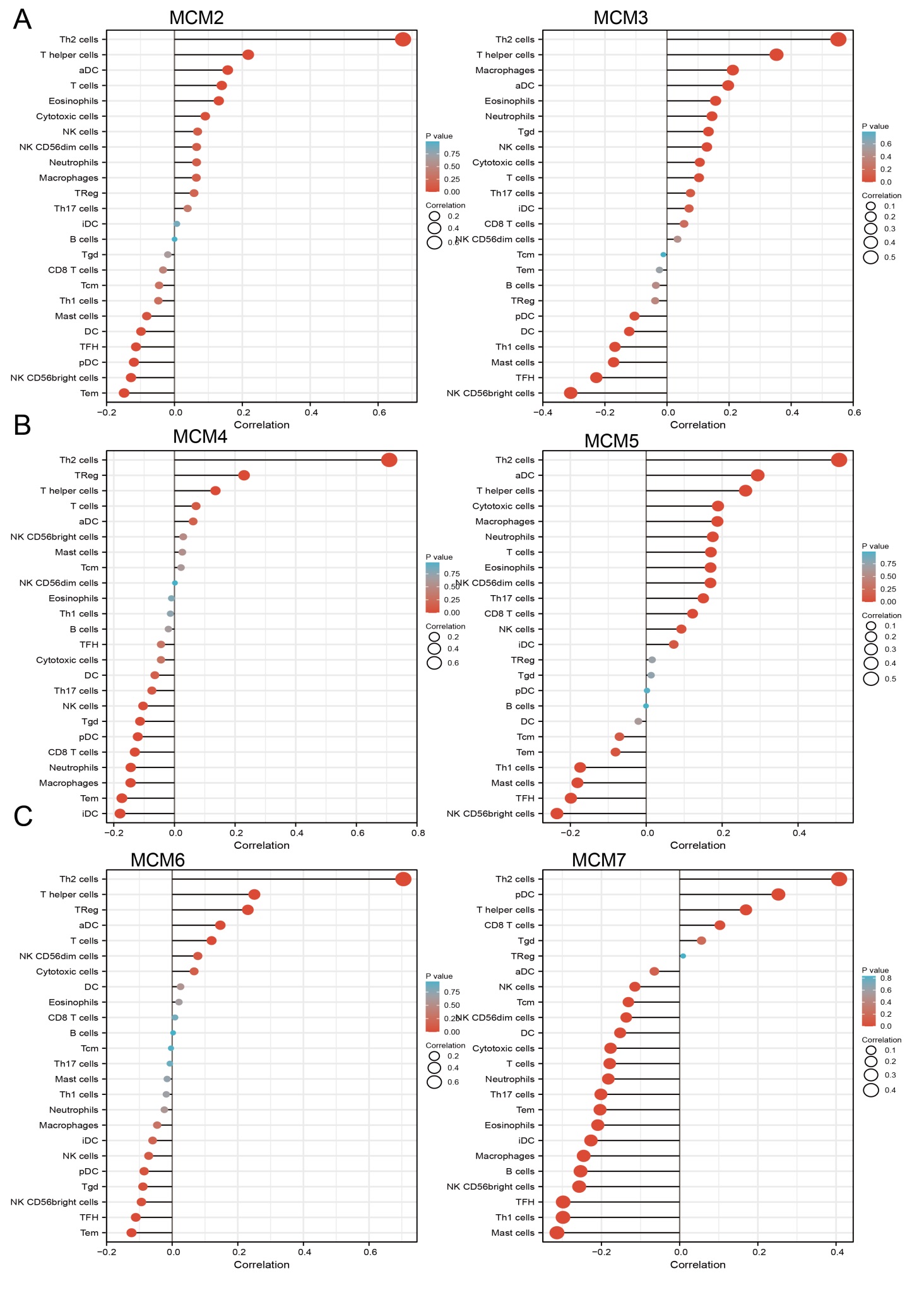


**Supplementary FIGURE 15 ⎜** **Analysis the correlation between the MCMs expression and immune cells infiltration.** (A-B)The correlation between MCMs expression and immune cells infiltration in LGG examine by the ssGSEA database. * P < 0.05, ** P < 0.01, *** P < 0.001.

**
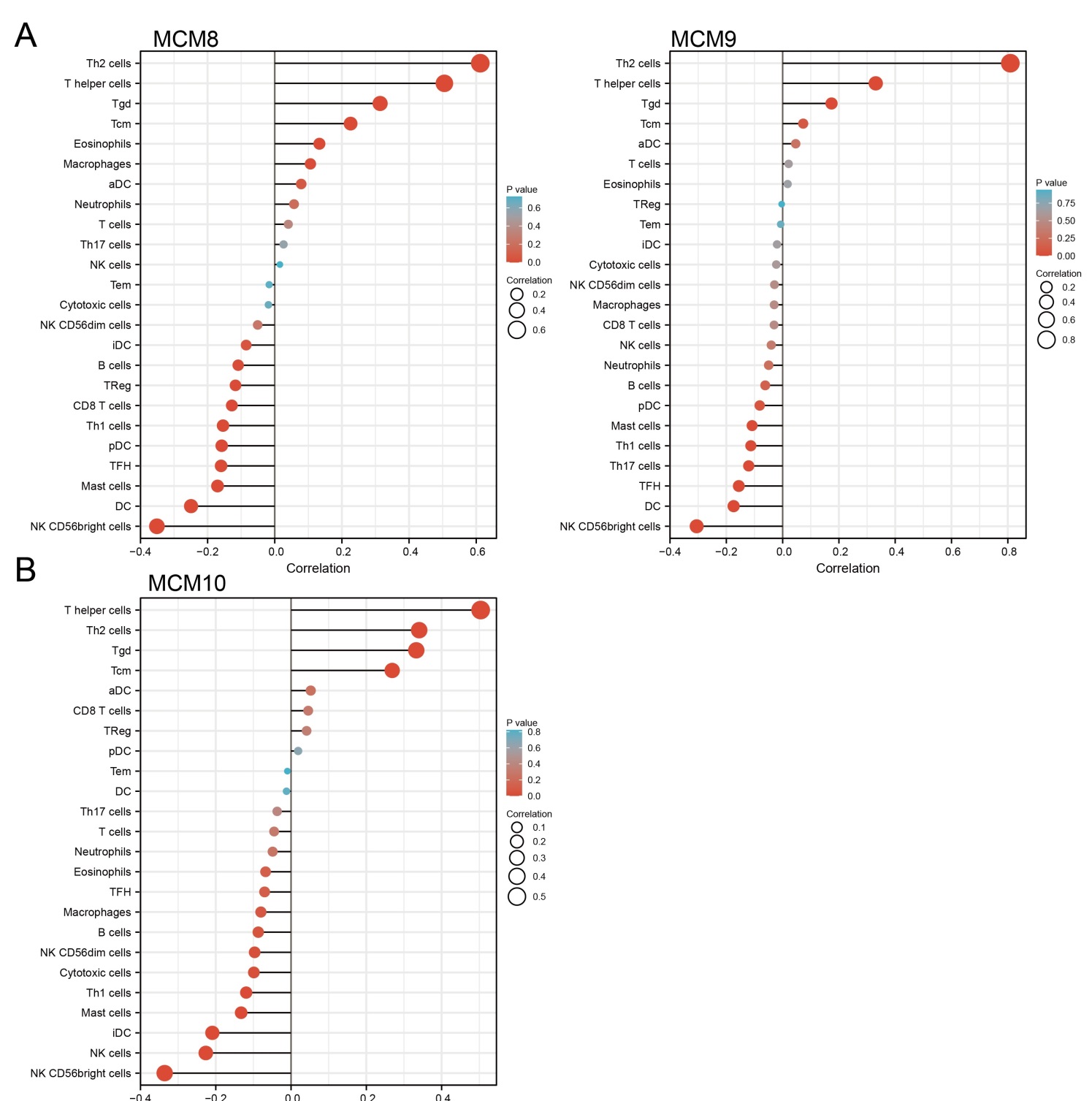
**

**Supplementary FIGURE 16 ⎜** **Analysis the correlation between the MCMs expression and immune cells infiltration.** (A-B)The correlation between MCMs expression and immune cells infiltration in LGG examine by the ssGSEA database. * P < 0.05, ** P < 0.01, *** P < 0.001.

**
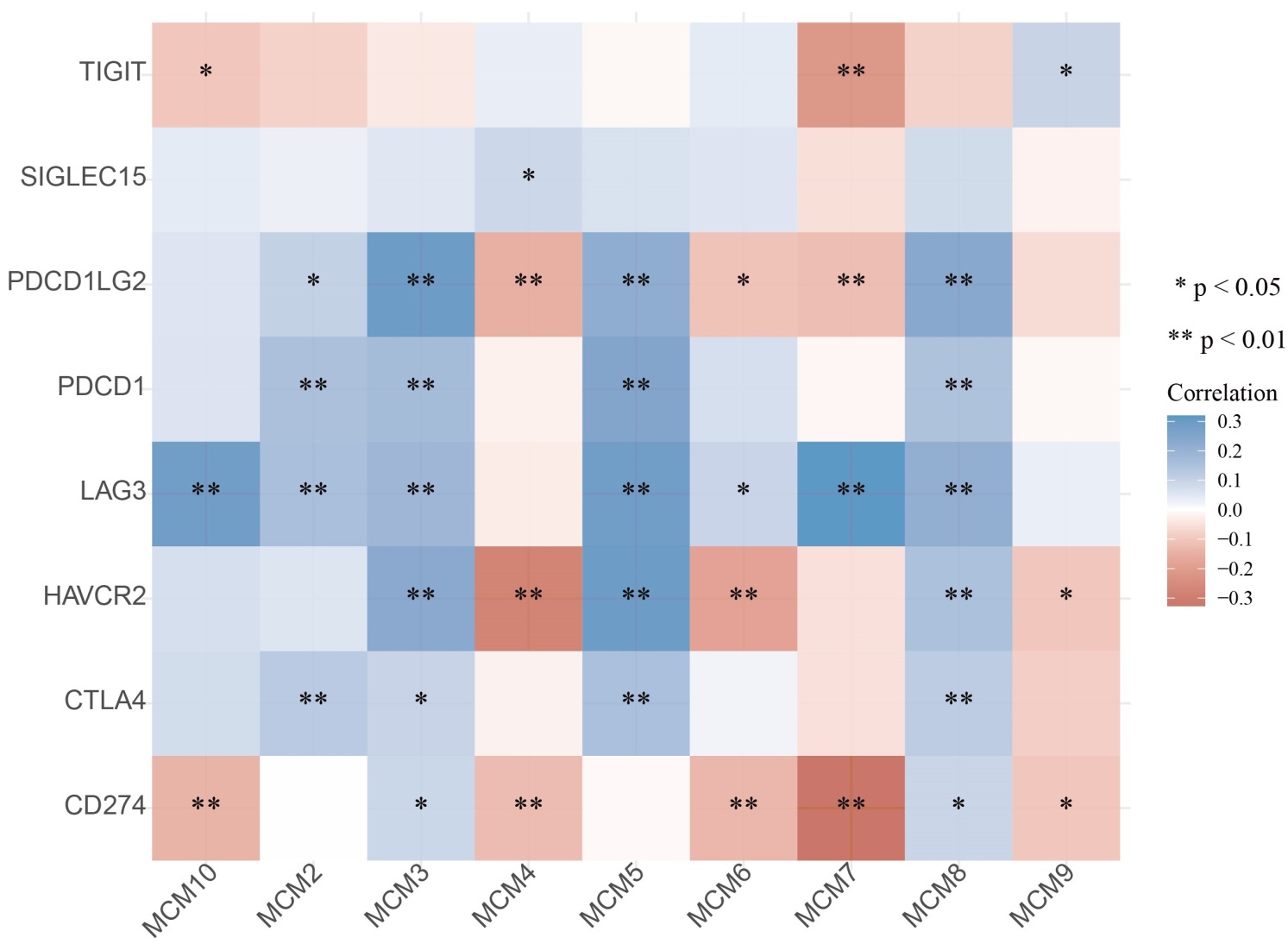
**

**Supplementary FIGURE 17 ⎜ Analysis the correlation between the MCMs expression and immune check points related gene.** Analysis the correlation between the MCMs expression and immune check points related gene in LGG analysis by TIMER database.

**
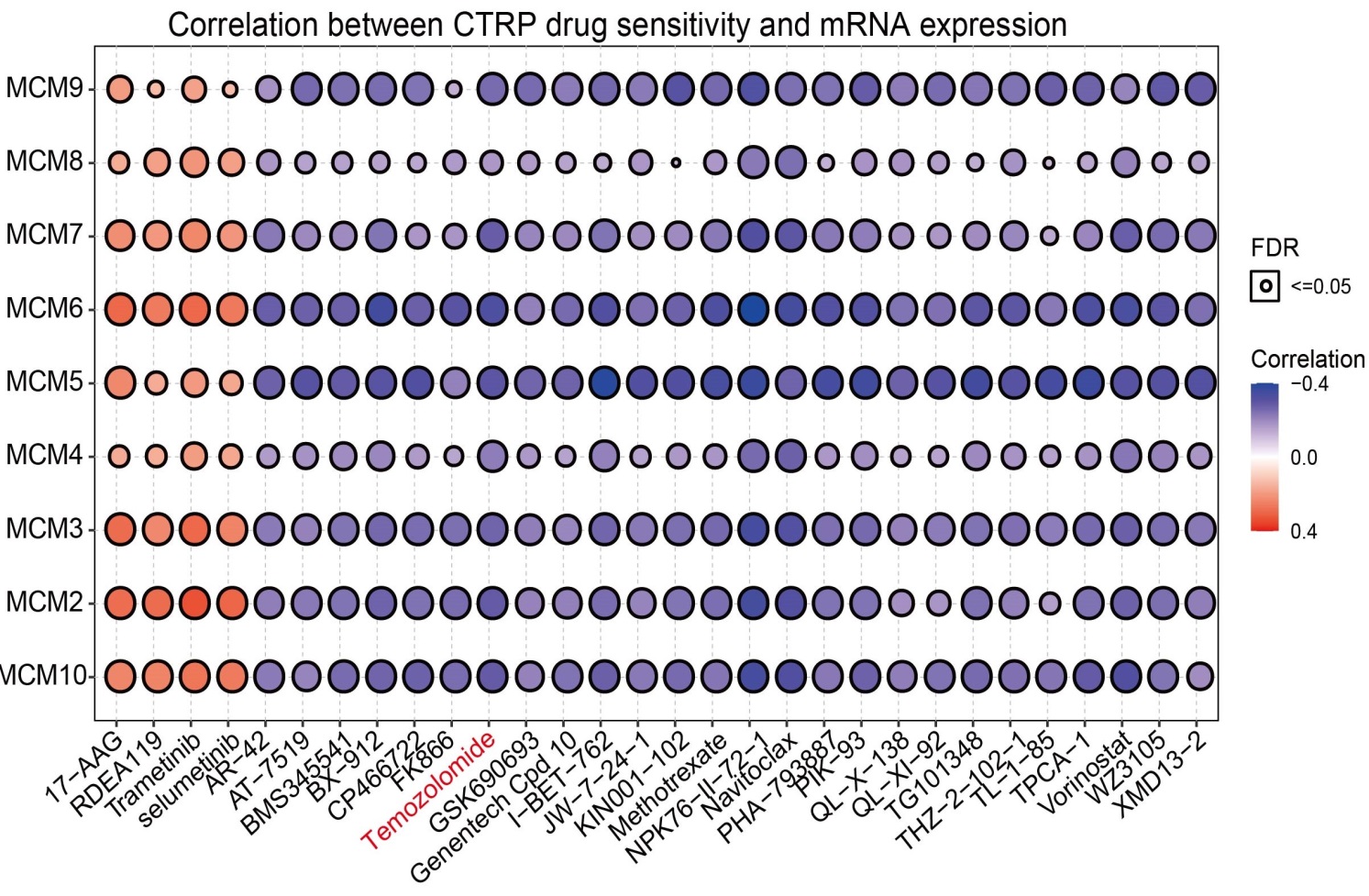
**

**Supplementary FIGURE 18  The correlation between MCMs expression and drug sensitivity by CTRP database.**

**
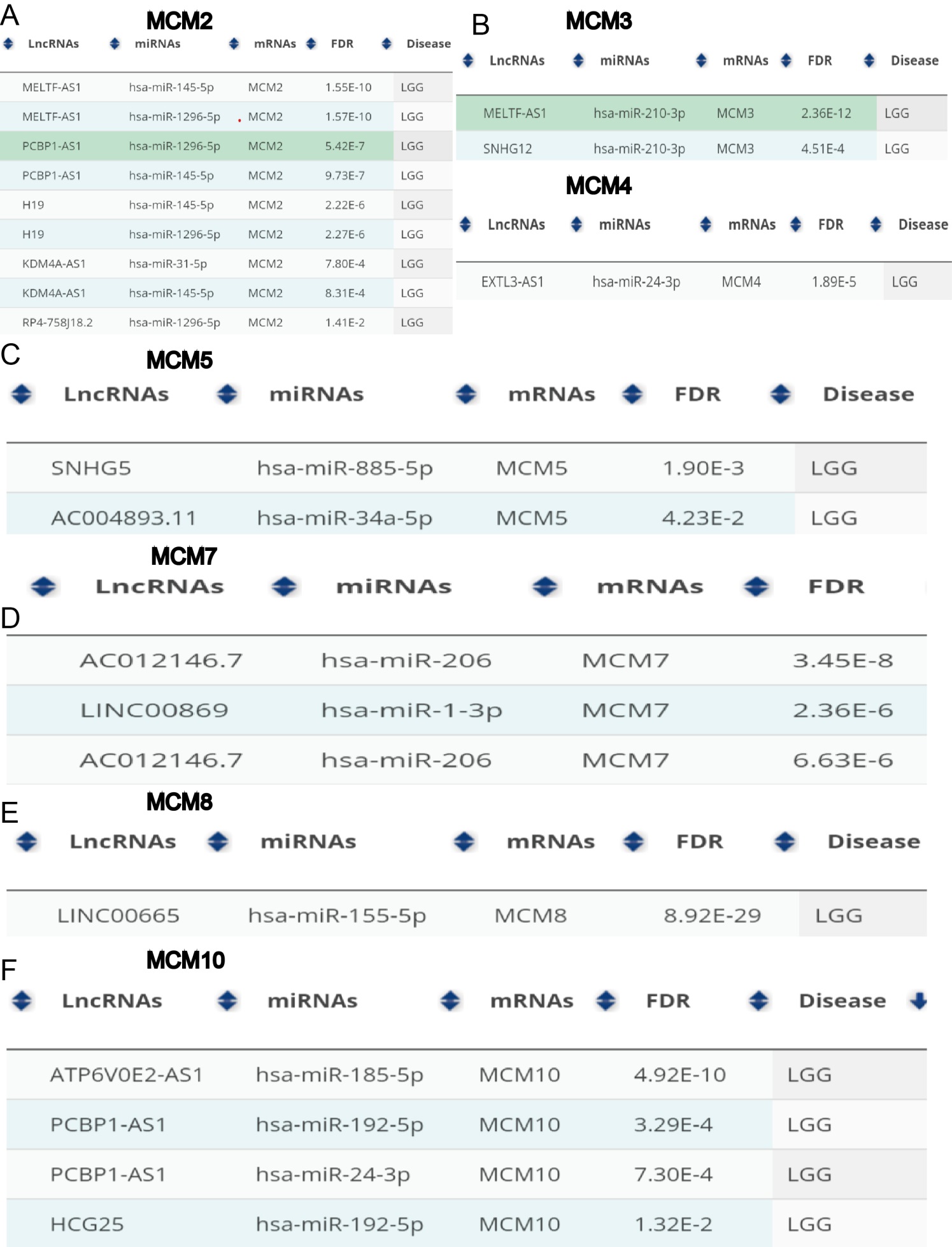
**

**Supplementary FIGURE 19 ⎜Analysis the ceRNA for the MCMs in LGG.** (A-F) Analysis the ceRNA network for the MCMs in LGG.
