# Supplementary material for: MCM4 is a novel prognostic biomarker and promotes cancer cell growth in glioma
Source: Front Oncol. 2022 Nov 17;12:1004324. doi: 10.3389/fonc.2022.1004324 (PMC9713251; doi:10.3389/fonc.2022.1004324)
Supplement: Supplementary file 2 [file DataSheet_2.docx]

**Figure 1A-1B R code**

library(tidyverse)

library(ggplot2)

library(reshape2)

library(car)

library(rstatix)

set.seed(100)

data <- data.frame(x = rnorm(100, 2, 1), y = rnorm(100, 1, 1))

data2 <- melt(data)

data3 <- lapply(data, function(x) get_summary_stats(data.frame(x)))

data3

# $x

# # A tibble: 1 x 13

# variable n min max median q1 q3 iqr mad mean sd se ci

# <chr> <dbl> <dbl> <dbl> <dbl> <dbl> <dbl> <dbl> <dbl> <dbl> <dbl> <dbl> <dbl>

# 1 x 100 -0.272 4.58 1.94 1.39 2.66 1.26 0.974 2.00 1.02 0.102 0.203

#

# $y

# # A tibble: 1 x 13

# variable n min max median q1 q3 iqr mad mean sd se ci

# <chr> <dbl> <dbl> <dbl> <dbl> <dbl> <dbl> <dbl> <dbl> <dbl> <dbl> <dbl> <dbl>

# 1 x 100 -1.14 3.17 0.927 0.568 1.45 0.878 0.648 1.01 0.796 0.08 0.158

data3 <- rbind(data3[[1]], data3[[2]])

data3[1] <- c("x", "y")

## Shapiro-Wilk normality test

lapply(data, function(x) shapiro.test(x))

# $x

#

# Shapiro-Wilk normality test

#

# data: x

# W = 0.98836, p-value = 0.535

#

#

# $y

#

# Shapiro-Wilk normality test

#

# data: x

# W = 0.98532, p-value = 0.3348

## Levene's Test

leveneTest(value~variable, data = data2)

# Levene's Test for Homogeneity of Variance (center = median)

# Df F value Pr(>F)

# group 1 4.4476 0.03621 *

# 198

# ---

# Signif. codes:

t.test(value~variable, data = data2, var.equal = T)

# Two Sample t-test

#

# data: value by variable

# t = 7.6613, df = 198, p-value = 8.012e-13

# alternative hypothesis: true difference in means is not equal to 0

# 95 percent confidence interval:

# 0.7364913 1.2470521

# sample estimates:

# mean in group x mean in group y

# 2.002913 1.011141

t.test(value~variable, data = data2, var.equal = F)

# Welch Two Sample t-test

#

# data: value by variable

# t = 7.6613, df = 186.92, p-value = 9.657e-13

# alternative hypothesis: true difference in means is not equal to 0

# 95 percent confidence interval:

# 0.7363983 1.2471452

# sample estimates:

# mean in group x mean in group y

# 2.002913 1.011141

wilcox.test(value~variable, data = data2)

# Wilcoxon rank sum test with continuity correction

#

# data: value by variable

# W = 7844, p-value = 3.711e-12

# alternative hypothesis: true location shift is not equal to 0

summary(aov(value~variable, data = data2))

# Df Sum Sq Mean Sq F value Pr(>F)

# variable 1 49.18 49.18 58.7 8.01e-13 ***

# Residuals 198 165.90 0.84

# ---

# Signif. codes:

ggplot(data2, aes(x = variable, y = value, color = variable, fill = variable)) +

geom_violin(alpha = 0.2) +

theme_bw()

ggplot(data2, aes(x = variable, y = value, color = variable, fill = variable)) +

geom_violin(alpha = 0.2) +

geom_point(position = position_jitter(0.3)) +

theme_bw()

ggplot(data2, aes(x = variable, y = value, color = variable, fill = variable)) +

geom_boxplot(alpha = 0.2) +

geom_point(position = position_jitter(0.3)) +

theme_bw()

ggplot(data2, aes(x = variable, y = value, color = variable, fill = variable)) +

geom_violin(alpha = 0.1) +

geom_boxplot(alpha = 0.1) +

geom_point(position = position_jitter(0.3)) +

theme_bw()

ggplot() +

geom_violin(data = data2, aes(x = variable, y = value, color = variable, fill = variable), alpha = 0.1) +

geom_errorbar(data = data3, aes(x = variable, ymin=mean-sd, ymax=mean+sd), width = 0.2)

**Figure 3-6 R code**

library(tidyverse)

library(ggplot2)

library(reshape2)

library(car)

library(rstatix)

set.seed(100)

data <- data.frame(x = rnorm(100, 2, 1), y = rnorm(100, 1, 1))

data2 <- melt(data)

data3 <- lapply(data, function(x) get_summary_stats(data.frame(x)))

data3

# $x

# # A tibble: 1 x 13

# variable n min max median q1 q3 iqr mad mean sd se ci

# <chr> <dbl> <dbl> <dbl> <dbl> <dbl> <dbl> <dbl> <dbl> <dbl> <dbl> <dbl> <dbl>

# 1 x 100 -0.272 4.58 1.94 1.39 2.66 1.26 0.974 2.00 1.02 0.102 0.203

#

# $y

# # A tibble: 1 x 13

# variable n min max median q1 q3 iqr mad mean sd se ci

# <chr> <dbl> <dbl> <dbl> <dbl> <dbl> <dbl> <dbl> <dbl> <dbl> <dbl> <dbl> <dbl>

# 1 x 100 -1.14 3.17 0.927 0.568 1.45 0.878 0.648 1.01 0.796 0.08 0.158

data3 <- rbind(data3[[1]], data3[[2]])

data3[1] <- c("x", "y")

## Shapiro-Wilk normality test

lapply(data, function(x) shapiro.test(x))

# $x

#

# Shapiro-Wilk normality test

#

# data: x

# W = 0.98836, p-value = 0.535

#

#

# $y

#

# Shapiro-Wilk normality test

#

# data: x

# W = 0.98532, p-value = 0.3348

## Levene's Test

leveneTest(value~variable, data = data2)

# Levene's Test for Homogeneity of Variance (center = median)

# Df F value Pr(>F)

# group 1 4.4476 0.03621 *

# 198

# ---

# Signif. codes:

t.test(value~variable, data = data2, var.equal = T)

# Two Sample t-test

#

# data: value by variable

# t = 7.6613, df = 198, p-value = 8.012e-13

# alternative hypothesis: true difference in means is not equal to 0

# 95 percent confidence interval:

# 0.7364913 1.2470521

# sample estimates:

# mean in group x mean in group y

# 2.002913 1.011141

t.test(value~variable, data = data2, var.equal = F)

# Welch Two Sample t-test

#

# data: value by variable

# t = 7.6613, df = 186.92, p-value = 9.657e-13

# alternative hypothesis: true difference in means is not equal to 0

# 95 percent confidence interval:

# 0.7363983 1.2471452

# sample estimates:

# mean in group x mean in group y

# 2.002913 1.011141

wilcox.test(value~variable, data = data2)

# Wilcoxon rank sum test with continuity correction

#

# data: value by variable

# W = 7844, p-value = 3.711e-12

# alternative hypothesis: true location shift is not equal to 0

summary(aov(value~variable, data = data2))

# Df Sum Sq Mean Sq F value Pr(>F)

# variable 1 49.18 49.18 58.7 8.01e-13 ***

# Residuals 198 165.90 0.84

# ---

# Signif. codes:

ggplot(data2, aes(x = variable, y = value, color = variable, fill = variable)) +

geom_violin(alpha = 0.2) +

theme_bw()

ggplot(data2, aes(x = variable, y = value, color = variable, fill = variable)) +

geom_violin(alpha = 0.2) +

geom_point(position = position_jitter(0.3)) +

theme_bw()

ggplot(data2, aes(x = variable, y = value, color = variable, fill = variable)) +

geom_boxplot(alpha = 0.2) +

geom_point(position = position_jitter(0.3)) +

theme_bw()

ggplot(data2, aes(x = variable, y = value, color = variable, fill = variable)) +

geom_violin(alpha = 0.1) +

geom_boxplot(alpha = 0.1) +

geom_point(position = position_jitter(0.3)) +

theme_bw()

ggplot() +

geom_violin(data = data2, aes(x = variable, y = value, color = variable, fill = variable), alpha = 0.1) +

geom_errorbar(data = data3, aes(x = variable, ymin=mean-sd, ymax=mean+sd), width = 0.2)

**Figure 7-9 R code**

if (!requireNamespace("survminer", quietly = TRUE))

install.packages("survminer")

library(survival)

library(survminer)

# data <- lung

# colnames(data)[5] <- "variable"

fit <- survfit(Surv(time, status) ~ variable, data = data)

print(fit)

# Call: survfit(formula = Surv(time, status) ~ variable, data = data)

#

# n events median 0.95LCL 0.95UCL

# variable=1 138 112 270 212 310

# variable=2 90 53 426 348 550

survdiff(Surv(time, status) ~ variable, data = data)

# survdiff(formula = Surv(time, status) ~ variable, data = data)

#

# N Observed Expected (O-E)^2/E (O-E)^2/V

# variable=1 138 112 91.6 4.55 10.3

# variable=2 90 53 73.4 5.68 10.3

#

# Chisq= 10.3 on 1 degrees of freedom, p= 0.001

fit2 <- coxph(Surv(time, status) ~ variable, data = data)

summary(fit2)

# Call:

# coxph(formula = Surv(time, status) ~ variable, data = data)

#

# n= 228, number of events= 165

#

# coef exp(coef) se(coef) z Pr(>|z|)

# variable -0.5310 0.5880 0.1672 -3.176 0.00149 **

# ---

# Signif. codes: #

# exp(coef) exp(-coef) lower .95 upper .95

# variable 0.588 1.701 0.4237 0.816

#

# Concordance= 0.579 (se = 0.021 )

# Likelihood ratio test= 10.63 on 1 df, p=0.001

# Wald test = 10.09 on 1 df, p=0.001

# Score (logrank) test = 10.33 on 1 df, p=0.001

# plot

ggsurvplot(fit = fit, data = data, pval = T)

**Figure 10 R code**

if (!requireNamespace("survminer", quietly = TRUE))

install.packages("survminer")

library(survival)

library(survminer)

# data <- lung

# colnames(data)[5] <- "variable"

fit <- survfit(Surv(time, status) ~ variable, data = data)

print(fit)

# Call: survfit(formula = Surv(time, status) ~ variable, data = data)

#

# n events median 0.95LCL 0.95UCL

# variable=1 138 112 270 212 310

# variable=2 90 53 426 348 550

survdiff(Surv(time, status) ~ variable, data = data)

# survdiff(formula = Surv(time, status) ~ variable, data = data)

#

# N Observed Expected (O-E)^2/E (O-E)^2/V

# variable=1 138 112 91.6 4.55 10.3

# variable=2 90 53 73.4 5.68 10.3

#

# Chisq= 10.3 on 1 degrees of freedom, p= 0.001

fit2 <- coxph(Surv(time, status) ~ variable, data = data)

summary(fit2)

# Call:

# coxph(formula = Surv(time, status) ~ variable, data = data)

#

# n= 228, number of events= 165

#

# coef exp(coef) se(coef) z Pr(>|z|)

# variable -0.5310 0.5880 0.1672 -3.176 0.00149 **

# ---

# Signif. codes: #

# exp(coef) exp(-coef) lower .95 upper .95

# variable 0.588 1.701 0.4237 0.816

#

# Concordance= 0.579 (se = 0.021 )

# Likelihood ratio test= 10.63 on 1 df, p=0.001

# Wald test = 10.09 on 1 df, p=0.001

# Score (logrank) test = 10.33 on 1 df, p=0.001

# plot

ggsurvplot(fit = fit, data = data, pval = T)

**Figure 11 R code**

if (!requireNamespace("survminer", quietly = TRUE))

install.packages("survminer")

library(survival)

library(survminer)

# data <- lung

# colnames(data)[5] <- "variable"

fit <- survfit(Surv(time, status) ~ variable, data = data)

print(fit)

# Call: survfit(formula = Surv(time, status) ~ variable, data = data)

#

# n events median 0.95LCL 0.95UCL

# variable=1 138 112 270 212 310

# variable=2 90 53 426 348 550

survdiff(Surv(time, status) ~ variable, data = data)

# survdiff(formula = Surv(time, status) ~ variable, data = data)

#

# N Observed Expected (O-E)^2/E (O-E)^2/V

# variable=1 138 112 91.6 4.55 10.3

# variable=2 90 53 73.4 5.68 10.3

#

# Chisq= 10.3 on 1 degrees of freedom, p= 0.001

fit2 <- coxph(Surv(time, status) ~ variable, data = data)

summary(fit2)

# Call:

# coxph(formula = Surv(time, status) ~ variable, data = data)

#

# n= 228, number of events= 165

#

# coef exp(coef) se(coef) z Pr(>|z|)

# variable -0.5310 0.5880 0.1672 -3.176 0.00149 **

# ---

# Signif. codes: #

# exp(coef) exp(-coef) lower .95 upper .95

# variable 0.588 1.701 0.4237 0.816

#

# Concordance= 0.579 (se = 0.021 )

# Likelihood ratio test= 10.63 on 1 df, p=0.001

# Wald test = 10.09 on 1 df, p=0.001

# Score (logrank) test = 10.33 on 1 df, p=0.001

# plot

ggsurvplot(fit = fit, data = data, pval = T)

**Figure 12 R code**

library(tidyverse)

library(survival)

library(readxl)

## read data

data <- read_xlsx("~/file.xlsx")

## tidy data

# data$event <- as.numeric(data$event)

# data$time <- as.numeric(data$time)

### numeric

data$Age <- as.numeric(data$Age)

data$Score <- as.numeric(data$Score)

### factor

data$Sex <- factor(data$Sex, levels = c("Male", "Female"))

data$Grade <- factor(data$Grade, levels = c("0", "1", "2"))

data$Stage <- factor(data$Stage, levels = c("Stage1", "Stage2", "Stage3", "Stage4"))

## summary

fit <- survfit(Surv(time, event) ~ Sex, data = data)

fit

# Call: survfit(formula = Surv(time, event) ~ Sex, data = data)

#

# n events median 0.95LCL 0.95UCL

# Sex=Female 90 53 426 348 550

# Sex=Male 138 112 270 212 310

## univariable Cox

fit <- coxph(Surv(time, event) ~ Sex, data = data)

summary(fit)

# Call:

# coxph(formula = Surv(time, event) ~ Sex, data = data)

#

# n= 228, number of events= 165

#

# coef exp(coef) se(coef) z Pr(>|z|)

# SexMale 0.5310 1.7007 0.1672 3.176 0.00149 **

# ---

# Signif. codes: #

# exp(coef) exp(-coef) lower .95 upper .95

# SexMale 1.701 0.588 1.226 2.36

#

# Concordance= 0.579 (se = 0.021 )

# Likelihood ratio test= 10.63 on 1 df, p=0.001

# Wald test = 10.09 on 1 df, p=0.001

# Score (logrank) test = 10.33 on 1 df, p=0.001

fit <- coxph(Surv(time, event) ~ Age, data = data)

summary(fit)

# Call:

# coxph(formula = Surv(time, event) ~ Age, data = data)

#

# n= 228, number of events= 165

#

# coef exp(coef) se(coef) z Pr(>|z|)

# Age 0.019543 1.019735 0.008735 2.237 0.0253 *

# ---

# Signif. codes: #

# exp(coef) exp(-coef) lower .95 upper .95

# Age 1.02 0.9806 1.002 1.037

#

# Concordance= 0.55 (se = 0.025 )

# Likelihood ratio test= 5.13 on 1 df, p=0.02

# Wald test = 5.01 on 1 df, p=0.03

# Score (logrank) test = 5.02 on 1 df, p=0.03

## multivariable Cox

fit <- coxph(Surv(time, event) ~ Age + Sex + Stage + Score, data = data)

summary(fit)

# Call:

# coxph(formula = Surv(time, event) ~ Age + Sex + Stage + Score,

# data = data)

#

# n= 224, number of events= 161

# (4 observations deleted due to missingness)

#

# coef exp(coef) se(coef) z Pr(>|z|)

# Age 0.010922 1.010982 0.008972 1.217 0.2235

# SexMale 0.536001 1.709159 0.169829 3.156 0.0016 **

# StageStage2 0.390113 1.477148 0.204289 1.910 0.0562 .

# StageStage3 0.693144 1.999993 0.283818 2.442 0.0146 *

# StageStage4 1.830114 6.234597 1.035948 1.767 0.0773 .

# Score -0.009346 0.990698 0.007127 -1.311 0.1897

# ---

# Signif. codes: #

# exp(coef) exp(-coef) lower .95 upper .95

# Age 1.0110 0.9891 0.9934 1.029

# SexMale 1.7092 0.5851 1.2252 2.384

# StageStage2 1.4771 0.6770 0.9898 2.205

# StageStage3 2.0000 0.5000 1.1467 3.488

# StageStage4 6.2346 0.1604 0.8185 47.491

# Score 0.9907 1.0094 0.9770 1.005

#

# Concordance= 0.654 (se = 0.025 )

# Likelihood ratio test= 32.28 on 6 df, p=1e-05

# Wald test = 32.66 on 6 df, p=1e-05

# Score (logrank) test = 34.96 on 6 df, p=4e-06
